# Supplementary material for: A low-background Tet-On system based on post-transcriptional regulation using Csy4
Source: PLoS One. 2020 Dec 30;15(12):e0244732. doi: 10.1371/journal.pone.0244732 (PMC7773235; doi:10.1371/journal.pone.0244732)
Supplement: S1 File — (DOCX) [file pone.0244732.s002.docx]

plasmid 1

CTAAATTGTAAGCGTTAATATTTTGTTAAAATTCGCGTTAAATTTTTGTTAAATCAGCTCATTTTTTAACCAATAGGCCGAAATCGGCAAAATCCCTTATAAATCAAAAGAATAGACCGAGATAGGGTTGAGTGTTGTTCCAGTTTGGAACAAGAGTCCACTATTAAAGAACGTGGACTCCAACGTCAAAGGGCGAAAAACCGTCTATCAGGGCGATGGCCCACTACGTGAACCATCACCCTAATCAAGTTTTTTGGGGTCGAGGTGCCGTAAAGCACTAAATCGGAACCCTAAAGGGAGCCCCCGATTTAGAGCTTGACGGGGAAAGCCGGCGAACGTGGCGAGAAAGGAAGGGAAGAAAGCGAAAGGAGCGGGCGCTAGGGCGCTGGCAAGTGTAGCGGTCACGCTGCGCGTAACCACCACACCCGCCGCGCTTAATGCGCCGCTACAGGGCGCGTCCCATTCGCCATTCAGGCTGCGCAACTGTTGGGAAGGGCGATCGGTGCGGGCCTCTTCGCTATTACGCCAGCTGGCGAAAGGGGGATGTGCTGCAAGGCGATTAAGTTGGGTAACGCCAGGGTTTTCCCAGTCACGACGTTGTAAAACGACGGCCAGTGAGCGCGCCTCGTTCATTCACGTTTTTGAACCCGTGGAGGACGGGCAGACTCGCGGTGCAAATGTGTTTTACAGCGTGATGGAGCAGATGAAGATGCTCGACACGCTGCAGAACACGCAGCTAGATTAACCCTAGAAAGATAATCATATTGTGACGTACGTTAAAGATAATCATGCGTAAAATTGACGCATGTGTTTTATCGGTCTGTATATCGAGGTTTATTTATTAATTTGAATAGATATTAAGTTTTATTATATTTACACTTACATACTAATAATAAATTCAACAAACAATTTATTTATGTTTATTTATTTATTAAAAAAAAACAAAAACTCAAAATTTCTTCTATAAAGTAACAAAACTTTTATCGAATTCatacgaaatgaactagtatcaagtgcggaaatttattaaaacacaatttatttcacaatacaattttattaagtaaagttataatgcaagtgtatatagggctacaatatagtatacatggattacaatgtgggtatacaaaagctatagtatggatataaaattattaagcacattggggttacaatataaacataatcaaccaCTTAAGtcacttgtacagctcgtccatgccgccggtggagtggcggccctcggcgcgttcgtactgttccacgatggtgtagtcctcgttgtgggaggtgatgtccaacttgatgttgacgttgtaggcgccgggcagctgcacgggcttcttggccttgtaggtggtcttgacctcagcgtcgtagtggccgccgtccttcagcttcagcctctgcttgatctcgcccttcagggcgccgtcctcggggtacatccgctcggaggaggcctcccagcccatggtTttcttctgcattacggggccgtcggaggggaagttggtgccgcgcagcttcaccttgtagatgaactcgccgtcctgcagggaggagtcctgggtcacggtcaccacgccgccgtcctcgaagttcatcacgcgctcccacttgaagccctcggggaaggacagcttcaagtagtcggggatgtcggcggggtgcttcacgtaggccttggagccgtacatgaactgaggggacaggatgtcccaggcgaagggcagggggccacccttggtcaccttcagcttggcggtctgggtgccctcgtaggggcggccctcgccctcgccctcgatctcgaactcgtggccgttcacggagccctccatgtgcaccttgaagcgcatgaactccttgatgatggccatgttatcctcctcgcccttgctcaccatggtggcaccggtcttgagtggagagagagagctagttataagcgtgtgtgtgcgctcttgtgactgaatcgaggagctaacacgggctcttatatacacaaatatgctccgcccacaacagcgattacaccatttctaggaataaatgagtaacaaggGGATCCcatgatgataaacaatgtatggtgctaatgttgcttcaacaacaattctgttgaactgtgttttcatgtttgccaacaagcacctttatactcggtggcctccccaccaccaacttttttgcactgcaaaaaaacacgcttttgcacgcgggcccatacatagtacaaactctacgtttcgtagactattttacataaatagtctacaccgttgtatacgctccaaatacactaccacacattgaacctttttgcagtgcaaaaaagtacgtgtcggcagtcacgtaggccggccttatcgggtcgcgtcctgtcacgtacgaatcacattatcggaccggacgagtgttgtcttatcgtgacaggacgccagcttcctgtgttgctaaccgcagccggacgcaactccttatcggaacaggacgcgcctccatatcagccgcgcgttatctcatgcgcgtgaccggacacgaggcgcccgtcccgcttatcgcgcctataaatacagcccgcaacgatctggtaaacacagttgaacagcatctgttacagcgacacaacGGTACCatccgcttaCgcGGCGCGCCtttgtattgtcatgttttaatacaatatgttatgtttaaatatgtttttaataaattttataaaataatttcaacttttattgtaacaacattgtccatttacacactcctttcaagcgcgtgcgcacccgaaaagcagggtcgccgctgacgcactgctaaaaatagcacgcgcctttcaaaagcgttcacacccgaaaCTCGAGGTCGACGGTATCGATAAGCTTGATATCTATAACAAGAAAATATATATATAATAAGTTATCACGTAAGTAGAACATGAAATAACAATATAATTATCGTATGAGTTAAATCTTAAAAGTCACGTAAAAGATAATCATGCGTCATTTTGACTCACGCGGTCGTTATAGTTCAAAATCAGTGACACTTACCGCATTGACAAGCACGCCTCACGGGAGCTCCAAGCGGCGACTGAGATGTCCTAAATGCACAGCGACGGATTCGCGCTATTTAGAAAGAGAGAGCAATATTTCAAGAATGCATGCGTCAATTTTACGCAGACTATCTTTCTAGGGTTAATCTAGCTGCATCAGGATCATATCGTCGGGTCTTTTTTCCGGCTCAGTCATCGCCCAAGCTGGCGCTATCTGGGCATCGGGGAGGAAGAAGCCCGTGCCTTTTCCCGCGAGGTTGAAGCGGCATGGAAAGAGTTTGCCGAGGATGACTGCTGCTGCATTGACGTTGAGCGAAAACGCACGTTTACCATGATGATTCGGGAAGGTGTGGCCATGCACGCCTTTAACGGTGAACTGTTCGTTCAGGCCACCTGGGATACCAGTTCGTCGCGGCTTTTCCGGACACAGTTCCGGATGGTCAGCCCGAAGCGCATCAGCAACCCGAACAATACCGGCGACAGCCGGAACTGCCGTGCCGGTGTGCAGATTAATGACAGCGGTGCGGCGCTGGGATATTACGTCAGCGAGGACGGGTATCCTGGCTGGATGCCGCAGAAATGGACATGGATACCCCGTGAGTTACCCGGCGGGCGCGCTTGGCGTAATCATGGTCATAGCTGTTTCCTGTGTGAAATTGTTATCCGCTCACAATTCCACACAACATACGAGCCGGAAGCATAAAGTGTAAAGCCTGGGGTGCCTAATGAGTGAGCTAACTCACATTAATTGCGTTGCGCTCACTGCCCGCTTTCCAGTCGGGAAACCTGTCGTGCCAGCTGCATTAATGAATCGGCCAACGCGCGGGGAGAGGCGGTTTGCGTATTGGGCGCTCTTCCGCTTCCTCGCTCACTGACTCGCTGCGCTCGGTCGTTCGGCTGCGGCGAGCGGTATCAGCTCACTCAAAGGCGGTAATACGGTTATCCACAGAATCAGGGGATAACGCAGGAAAGAACATGTGAGCAAAAGGCCAGCAAAAGGCCAGGAACCGTAAAAAGGCCGCGTTGCTGGCGTTTTTCCATAGGCTCCGCCCCCCTGACGAGCATCACAAAAATCGACGCTCAAGTCAGAGGTGGCGAAACCCGACAGGACTATAAAGATACCAGGCGTTTCCCCCTGGAAGCTCCCTCGTGCGCTCTCCTGTTCCGACCCTGCCGCTTACCGGATACCTGTCCGCCTTTCTCCCTTCGGGAAGCGTGGCGCTTTCTCATAGCTCACGCTGTAGGTATCTCAGTTCGGTGTAGGTCGTTCGCTCCAAGCTGGGCTGTGTGCACGAACCCCCCGTTCAGCCCGACCGCTGCGCCTTATCCGGTAACTATCGTCTTGAGTCCAACCCGGTAAGACACGACTTATCGCCACTGGCAGCAGCCACTGGTAACAGGATTAGCAGAGCGAGGTATGTAGGCGGTGCTACAGAGTTCTTGAAGTGGTGGCCTAACTACGGCTACACTAGAAGGACAGTATTTGGTATCTGCGCTCTGCTGAAGCCAGTTACCTTCGGAAAAAGAGTTGGTAGCTCTTGATCCGGCAAACAAACCACCGCTGGTAGCGGTGGTTTTTTTGTTTGCAAGCAGCAGATTACGCGCAGAAAAAAAGGATCTCAAGAAGATCCTTTGATCTTTTCTACGGGGTCTGACGCTCAGTGGAACGAAAACTCACGTTAAGGGATTTTGGTCATGAGATTATCAAAAAGGATCTTCACCTAGATCCTTTTAAATTAAAAATGAAGTTTTAAATCAATCTAAAGTATATATGAGTAAACTTGGTCTGACAGTTACCAATGCTTAATCAGTGAGGCACCTATCTCAGCGATCTGTCTATTTCGTTCATCCATAGTTGCCTGACTCCCCGTCGTGTAGATAACTACGATACGGGAGGGCTTACCATCTGGCCCCAGTGCTGCAATGATACCGCGAGACCCACGCTCACCGGCTCCAGATTTATCAGCAATAAACCAGCCAGCCGGAAGGGCCGAGCGCAGAAGTGGTCCTGCAACTTTATCCGCCTCCATCCAGTCTATTAATTGTTGCCGGGAAGCTAGAGTAAGTAGTTCGCCAGTTAATAGTTTGCGCAACGTTGTTGCCATTGCTACAGGCATCGTGGTGTCACGCTCGTCGTTTGGTATGGCTTCATTCAGCTCCGGTTCCCAACGATCAAGGCGAGTTACATGATCCCCCATGTTGTGCAAAAAAGCGGTTAGCTCCTTCGGTCCTCCGATCGTTGTCAGAAGTAAGTTGGCCGCAGTGTTATCACTCATGGTTATGGCAGCACTGCATAATTCTCTTACTGTCATGCCATCCGTAAGATGCTTTTCTGTGACTGGTGAGTACTCAACCAAGTCATTCTGAGAATAGTGTATGCGGCGACCGAGTTGCTCTTGCCCGGCGTCAATACGGGATAATACCGCGCCACATAGCAGAACTTTAAAAGTGCTCATCATTGGAAAACGTTCTTCGGGGCGAAAACTCTCAAGGATCTTACCGCTGTTGAGATCCAGTTCGATGTAACCCACTCGTGCACCCAACTGATCTTCAGCATCTTTTACTTTCACCAGCGTTTCTGGGTGAGCAAAAACAGGAAGGCAAAATGCCGCAAAAAAGGGAATAAGGGCGACACGGAAATGTTGAATACTCATACTCTTCCTTTTTCAATATTATTGAAGCATTTATCAGGGTTATTGTCTCATGAGCGGATACATATTTGAATGTATTTAGAAAAATAAACAAATAGGGGTTCCGCGCACATTTCCCCGAAAAGTGCCAC

plasmid 2

CTAAATTGTAAGCGTTAATATTTTGTTAAAATTCGCGTTAAATTTTTGTTAAATCAGCTCATTTTTTAACCAATAGGCCGAAATCGGCAAAATCCCTTATAAATCAAAAGAATAGACCGAGATAGGGTTGAGTGTTGTTCCAGTTTGGAACAAGAGTCCACTATTAAAGAACGTGGACTCCAACGTCAAAGGGCGAAAAACCGTCTATCAGGGCGATGGCCCACTACGTGAACCATCACCCTAATCAAGTTTTTTGGGGTCGAGGTGCCGTAAAGCACTAAATCGGAACCCTAAAGGGAGCCCCCGATTTAGAGCTTGACGGGGAAAGCCGGCGAACGTGGCGAGAAAGGAAGGGAAGAAAGCGAAAGGAGCGGGCGCTAGGGCGCTGGCAAGTGTAGCGGTCACGCTGCGCGTAACCACCACACCCGCCGCGCTTAATGCGCCGCTACAGGGCGCGTCCCATTCGCCATTCAGGCTGCGCAACTGTTGGGAAGGGCGATCGGTGCGGGCCTCTTCGCTATTACGCCAGCTGGCGAAAGGGGGATGTGCTGCAAGGCGATTAAGTTGGGTAACGCCAGGGTTTTCCCAGTCACGACGTTGTAAAACGACGGCCAGTGAGCGCGCCTCGTTCATTCACGTTTTTGAACCCGTGGAGGACGGGCAGACTCGCGGTGCAAATGTGTTTTACAGCGTGATGGAGCAGATGAAGATGCTCGACACGCTGCAGAACACGCAGCTAGATTAACCCTAGAAAGATAATCATATTGTGACGTACGTTAAAGATAATCATGCGTAAAATTGACGCATGTGTTTTATCGGTCTGTATATCGAGGTTTATTTATTAATTTGAATAGATATTAAGTTTTATTATATTTACACTTACATACTAATAATAAATTCAACAAACAATTTATTTATGTTTATTTATTTATTAAAAAAAAACAAAAACTCAAAATTTCTTCTATAAAGTAACAAAACTTTTATCGAATTCatacgaaatgaactagtatcaagtgcggaaatttattaaaacacaatttatttcacaatacaattttattaagtaaagttataatgcaagtgtatatagggctacaatatagtatacatggattacaatgtgggtatacaaaagctatagtatggatataaaattattaagcacattggggttacaatataaacataatcaaccaCTTAAGtcacttgtacagctcgtccatgccgccggtggagtggcggccctcggcgcgttcgtactgttccacgatggtgtagtcctcgttgtgggaggtgatgtccaacttgatgttgacgttgtaggcgccgggcagctgcacgggcttcttggccttgtaggtggtcttgacctcagcgtcgtagtggccgccgtccttcagcttcagcctctgcttgatctcgcccttcagggcgccgtcctcggggtacatccgctcggaggaggcctcccagcccatggtTttcttctgcattacggggccgtcggaggggaagttggtgccgcgcagcttcaccttgtagatgaactcgccgtcctgcagggaggagtcctgggtcacggtcaccacgccgccgtcctcgaagttcatcacgcgctcccacttgaagccctcggggaaggacagcttcaagtagtcggggatgtcggcggggtgcttcacgtaggccttggagccgtacatgaactgaggggacaggatgtcccaggcgaagggcagggggccacccttggtcaccttcagcttggcggtctgggtgccctcgtaggggcggccctcgccctcgccctcgatctcgaactcgtggccgttcacggagccctccatgtgcaccttgaagcgcatgaactccttgatgatggccatgttatcctcctcgcccttgctcaccatggtggcaccggtcttgagtggagagagagagctagttataagcgtgtgtgtgcgctcttgtgactgaatcgaggagctaacacgggctcttatatacacaaatatgctccgcccacaacagcgattacaccatttctaggaataaatgagtaacaaggGGATCCcatgatgataaacaatgtatggtgctaatgttgcttcaacaacaattctgttgaactgtgttttcatgtttgccaacaagcacctttatactcggtggcctccccaccaccaacttttttgcactgcaaaaaaacacgcttttgcacgcgggcccatacatagtacaaactctacgtttcgtagactattttacataaatagtctacaccgttgtatacgctccaaatacactaccacacattgaacctttttgcagtgcaaaaaagtacgtgtcggcagtcacgtaggccggccttatcgggtcgcgtcctgtcacgtacgaatcacattatcggaccggacgagtgttgtcttatcgtgacaggacgccagcttcctgtgttgctaaccgcagccggacgcaactccttatcggaacaggacgcgcctccatatcagccgcgcgttatctcatgcgcgtgaccggacacgaggcgcccgtcccgcttatcgcgcctataaatacagcccgcaacgatctggtaaacacagttgaacagcatctgttacagcgacacaacGGTACCgccACCatggaaagaggggaaatctggcttgtctcgcttgatcctaccgcaggtcatgagcagcagggaacgcggccggtgctgattgtcacaccggcggcctttaatcgcgtgacccgcctgcctgttgttgtgcccgtaaccagcggaggcaattttgcccgcactgccggctttgcggtgtcgttggatggtgttggcatacgtaccacaggtgttgtacgttgcgatcaaccccggacaattgatatgaaagcacggggcggaaaacgactcgaacgggttccggagactatcatgaacgaagttcttggccgcctgtccactattctgacttgaGGCGCGCCtttgtattgtcatgttttaatacaatatgttatgtttaaatatgtttttaataaattttataaaataatttcaacttttattgtaacaacattgtccatttacacactcctttcaagcgcgtgcgcacccgaaaagcagggtcgccgctgacgcactgctaaaaatagcacgcgcctttcaaaagcgttcacacccgaaaCTCGAGGTCGACGGTATCGATAAGCTTGATATCTATAACAAGAAAATATATATATAATAAGTTATCACGTAAGTAGAACATGAAATAACAATATAATTATCGTATGAGTTAAATCTTAAAAGTCACGTAAAAGATAATCATGCGTCATTTTGACTCACGCGGTCGTTATAGTTCAAAATCAGTGACACTTACCGCATTGACAAGCACGCCTCACGGGAGCTCCAAGCGGCGACTGAGATGTCCTAAATGCACAGCGACGGATTCGCGCTATTTAGAAAGAGAGAGCAATATTTCAAGAATGCATGCGTCAATTTTACGCAGACTATCTTTCTAGGGTTAATCTAGCTGCATCAGGATCATATCGTCGGGTCTTTTTTCCGGCTCAGTCATCGCCCAAGCTGGCGCTATCTGGGCATCGGGGAGGAAGAAGCCCGTGCCTTTTCCCGCGAGGTTGAAGCGGCATGGAAAGAGTTTGCCGAGGATGACTGCTGCTGCATTGACGTTGAGCGAAAACGCACGTTTACCATGATGATTCGGGAAGGTGTGGCCATGCACGCCTTTAACGGTGAACTGTTCGTTCAGGCCACCTGGGATACCAGTTCGTCGCGGCTTTTCCGGACACAGTTCCGGATGGTCAGCCCGAAGCGCATCAGCAACCCGAACAATACCGGCGACAGCCGGAACTGCCGTGCCGGTGTGCAGATTAATGACAGCGGTGCGGCGCTGGGATATTACGTCAGCGAGGACGGGTATCCTGGCTGGATGCCGCAGAAATGGACATGGATACCCCGTGAGTTACCCGGCGGGCGCGCTTGGCGTAATCATGGTCATAGCTGTTTCCTGTGTGAAATTGTTATCCGCTCACAATTCCACACAACATACGAGCCGGAAGCATAAAGTGTAAAGCCTGGGGTGCCTAATGAGTGAGCTAACTCACATTAATTGCGTTGCGCTCACTGCCCGCTTTCCAGTCGGGAAACCTGTCGTGCCAGCTGCATTAATGAATCGGCCAACGCGCGGGGAGAGGCGGTTTGCGTATTGGGCGCTCTTCCGCTTCCTCGCTCACTGACTCGCTGCGCTCGGTCGTTCGGCTGCGGCGAGCGGTATCAGCTCACTCAAAGGCGGTAATACGGTTATCCACAGAATCAGGGGATAACGCAGGAAAGAACATGTGAGCAAAAGGCCAGCAAAAGGCCAGGAACCGTAAAAAGGCCGCGTTGCTGGCGTTTTTCCATAGGCTCCGCCCCCCTGACGAGCATCACAAAAATCGACGCTCAAGTCAGAGGTGGCGAAACCCGACAGGACTATAAAGATACCAGGCGTTTCCCCCTGGAAGCTCCCTCGTGCGCTCTCCTGTTCCGACCCTGCCGCTTACCGGATACCTGTCCGCCTTTCTCCCTTCGGGAAGCGTGGCGCTTTCTCATAGCTCACGCTGTAGGTATCTCAGTTCGGTGTAGGTCGTTCGCTCCAAGCTGGGCTGTGTGCACGAACCCCCCGTTCAGCCCGACCGCTGCGCCTTATCCGGTAACTATCGTCTTGAGTCCAACCCGGTAAGACACGACTTATCGCCACTGGCAGCAGCCACTGGTAACAGGATTAGCAGAGCGAGGTATGTAGGCGGTGCTACAGAGTTCTTGAAGTGGTGGCCTAACTACGGCTACACTAGAAGGACAGTATTTGGTATCTGCGCTCTGCTGAAGCCAGTTACCTTCGGAAAAAGAGTTGGTAGCTCTTGATCCGGCAAACAAACCACCGCTGGTAGCGGTGGTTTTTTTGTTTGCAAGCAGCAGATTACGCGCAGAAAAAAAGGATCTCAAGAAGATCCTTTGATCTTTTCTACGGGGTCTGACGCTCAGTGGAACGAAAACTCACGTTAAGGGATTTTGGTCATGAGATTATCAAAAAGGATCTTCACCTAGATCCTTTTAAATTAAAAATGAAGTTTTAAATCAATCTAAAGTATATATGAGTAAACTTGGTCTGACAGTTACCAATGCTTAATCAGTGAGGCACCTATCTCAGCGATCTGTCTATTTCGTTCATCCATAGTTGCCTGACTCCCCGTCGTGTAGATAACTACGATACGGGAGGGCTTACCATCTGGCCCCAGTGCTGCAATGATACCGCGAGACCCACGCTCACCGGCTCCAGATTTATCAGCAATAAACCAGCCAGCCGGAAGGGCCGAGCGCAGAAGTGGTCCTGCAACTTTATCCGCCTCCATCCAGTCTATTAATTGTTGCCGGGAAGCTAGAGTAAGTAGTTCGCCAGTTAATAGTTTGCGCAACGTTGTTGCCATTGCTACAGGCATCGTGGTGTCACGCTCGTCGTTTGGTATGGCTTCATTCAGCTCCGGTTCCCAACGATCAAGGCGAGTTACATGATCCCCCATGTTGTGCAAAAAAGCGGTTAGCTCCTTCGGTCCTCCGATCGTTGTCAGAAGTAAGTTGGCCGCAGTGTTATCACTCATGGTTATGGCAGCACTGCATAATTCTCTTACTGTCATGCCATCCGTAAGATGCTTTTCTGTGACTGGTGAGTACTCAACCAAGTCATTCTGAGAATAGTGTATGCGGCGACCGAGTTGCTCTTGCCCGGCGTCAATACGGGATAATACCGCGCCACATAGCAGAACTTTAAAAGTGCTCATCATTGGAAAACGTTCTTCGGGGCGAAAACTCTCAAGGATCTTACCGCTGTTGAGATCCAGTTCGATGTAACCCACTCGTGCACCCAACTGATCTTCAGCATCTTTTACTTTCACCAGCGTTTCTGGGTGAGCAAAAACAGGAAGGCAAAATGCCGCAAAAAAGGGAATAAGGGCGACACGGAAATGTTGAATACTCATACTCTTCCTTTTTCAATATTATTGAAGCATTTATCAGGGTTATTGTCTCATGAGCGGATACATATTTGAATGTATTTAGAAAAATAAACAAATAGGGGTTCCGCGCACATTTCCCCGAAAAGTGCCAC

plasmid 3

CTAAATTGTAAGCGTTAATATTTTGTTAAAATTCGCGTTAAATTTTTGTTAAATCAGCTCATTTTTTAACCAATAGGCCGAAATCGGCAAAATCCCTTATAAATCAAAAGAATAGACCGAGATAGGGTTGAGTGTTGTTCCAGTTTGGAACAAGAGTCCACTATTAAAGAACGTGGACTCCAACGTCAAAGGGCGAAAAACCGTCTATCAGGGCGATGGCCCACTACGTGAACCATCACCCTAATCAAGTTTTTTGGGGTCGAGGTGCCGTAAAGCACTAAATCGGAACCCTAAAGGGAGCCCCCGATTTAGAGCTTGACGGGGAAAGCCGGCGAACGTGGCGAGAAAGGAAGGGAAGAAAGCGAAAGGAGCGGGCGCTAGGGCGCTGGCAAGTGTAGCGGTCACGCTGCGCGTAACCACCACACCCGCCGCGCTTAATGCGCCGCTACAGGGCGCGTCCCATTCGCCATTCAGGCTGCGCAACTGTTGGGAAGGGCGATCGGTGCGGGCCTCTTCGCTATTACGCCAGCTGGCGAAAGGGGGATGTGCTGCAAGGCGATTAAGTTGGGTAACGCCAGGGTTTTCCCAGTCACGACGTTGTAAAACGACGGCCAGTGAGCGCGCCTCGTTCATTCACGTTTTTGAACCCGTGGAGGACGGGCAGACTCGCGGTGCAAATGTGTTTTACAGCGTGATGGAGCAGATGAAGATGCTCGACACGCTGCAGAACACGCAGCTAGATTAACCCTAGAAAGATAATCATATTGTGACGTACGTTAAAGATAATCATGCGTAAAATTGACGCATGTGTTTTATCGGTCTGTATATCGAGGTTTATTTATTAATTTGAATAGATATTAAGTTTTATTATATTTACACTTACATACTAATAATAAATTCAACAAACAATTTATTTATGTTTATTTATTTATTAAAAAAAAACAAAAACTCAAAATTTCTTCTATAAAGTAACAAAACTTTTATCGAATTCatacgaaatgaactagtatcaagtgcggaaatttattaaaacacaatttatttcacaatacaattttattaagtaaagttataatgcaagtgtatatagggctacaatatagtatacatggattacaatgtgggtatacaaaagctatagtatggatataaaattattaagcacattggggttacaatataaacataatcaaccaCTTAAGtcacttgtacagctcgtccatgccgccggtggagtggcggccctcggcgcgttcgtactgttccacgatggtgtagtcctcgttgtgggaggtgatgtccaacttgatgttgacgttgtaggcgccgggcagctgcacgggcttcttggccttgtaggtggtcttgacctcagcgtcgtagtggccgccgtccttcagcttcagcctctgcttgatctcgcccttcagggcgccgtcctcggggtacatccgctcggaggaggcctcccagcccatggtTttcttctgcattacggggccgtcggaggggaagttggtgccgcgcagcttcaccttgtagatgaactcgccgtcctgcagggaggagtcctgggtcacggtcaccacgccgccgtcctcgaagttcatcacgcgctcccacttgaagccctcggggaaggacagcttcaagtagtcggggatgtcggcggggtgcttcacgtaggccttggagccgtacatgaactgaggggacaggatgtcccaggcgaagggcagggggccacccttggtcaccttcagcttggcggtctgggtgccctcgtaggggcggccctcgccctcgccctcgatctcgaactcgtggccgttcacggagccctccatgtgcaccttgaagcgcatgaactccttgatgatggccatgttatcctcctcgcccttgctcaccatggtggcaccggtcttgagtggagagagagagctagttataagcgtgtgtgtgcgctcttgtgactgaatcgaggagctaacacgggctcttatatacacaaatatgctccgcccacaacagcgattacaccatttctaggaataaatgagtaacaaggGGATCCcatgatgataaacaatgtatggtgctaatgttgcttcaacaacaattctgttgaactgtgttttcatgtttgccaacaagcacctttatactcggtggcctccccaccaccaacttttttgcactgcaaaaaaacacgcttttgcacgcgggcccatacatagtacaaactctacgtttcgtagactattttacataaatagtctacaccgttgtatacgctccaaatacactaccacacattgaacctttttgcagtgcaaaaaagtacgtgtcggcagtcacgtaggccggccttatcgggtcgcgtcctgtcacgtacgaatcacattatcggaccggacgagtgttgtcttatcgtgacaggacgccagcttcctgtgttgctaaccgcagccggacgcaactccttatcggaacaggacgcgcctccatatcagccgcgcgttatctcatgcgcgtgaccggacacgaggcgcccgtcccgcttatcgcgcctataaatacagcccgcaacgatctggtaaacacagttgaacagcatctgttacagcgacacaacGGTACCgccACCatgtctagactggacaagagcaaagtcataaactctgctctggaattactcaatggagtcggtatcgaaggcctgacgacaaggaaactcgctcaaaagctgggagttgagcagcctaccctgtactggcacgtgaagaacaagcgggccctgctcgatgccctgccaatcgagatgctggacaggcatcatacccactcctgccccctggaaggcgagtcatggcaagactttctgcggaacaacgccaagtcataccgctgtgctctcctctcacatcgcgacggggctaaagtgcatctcggcacccgcccaacagagaaacagtacgaaaccctggaaaatcagctcgcgttcctgtgtcagcaaggcttctccctggagaacgcactgtacgctctgtccgccgtgggccactttacactgggctgcgtattggaggaacaggagcatcaagtagcaaaagaggaaagagagacacctaccaccgattctatgcccccacttctgaaacaagcaattgagctgttcgaccggcagggagccgaacctgccttccttttcggcctggaactaatcatatgtggcctggagaaacagctaaagtgcgaaagcggcgggccgaccgacgcccttgacgattttgacttagacatgctcccagccgatgcccttgacgactttgaccttgatatgctgcctgctgacgctcttgacgattttgaccttgacatgctccccgggtaaGGCGCGCCtttgtattgtcatgttttaatacaatatgttatgtttaaatatgtttttaataaattttataaaataatttcaacttttattgtaacaacattgtccatttacacactcctttcaagcgcgtgcgcacccgaaaagcagggtcgccgctgacgcactgctaaaaatagcacgcgcctttcaaaagcgttcacacccgaaaCTCGAGGTAAGATACATTGATGAGTTTGGACAAACCACAACTAGAATGCAGTGAAAAAAATGCTTTATTTGTGAAATTTGTGATGCTATTGCTTTATTTGTAACCATTATAAGCTGCAATAAACAAGTTAACAACAACAATTGCATTCATTTTATGTTTCAGGTTCAGGGGGAGGTGTGGGAGGTTTTTTAAAGCAAGTAAAACCTCTACAAATGTGGTATGGCTGATTATGATCGTCGACtcaagtcagaatagtggacaggcggccaagaacttcgttcatgatagtctccggaacccgttcgagtcgttttccgccccgtgctttcatatcaattgtccggggttgatcgcaacgtacaacacctgtggtacgtatgccaacaccatccaacgacaccgcaaagccggcagtgcgggcaaaattgcctccgctggttacgggcacaacaacaggcaggcgggtcacgcgattaaaggccgccggtgtgacaatcagcaccggccgcgttccctgctgctcatgacctgcggtaggatcaagcgagacaagccagatttcccctctttccatggtgGCGGCCGCaagtataagacaaaagtgttgtggaattgctccaggcgatctgacggttcactaaacgagctctgcttttataggcgccCACCGTACACGCCTAAAGCTTATACGTtctctatcactgatagggagtaaactggatatacgttctctatcactgatagggagtaaactgtagatacgttctctatcactgatagggagtaaactggtcatacgttctctatcactgatagggagtaaactccttatacgttctctatcactgatagggagtaaagtctgcatacgttctctatcactgatagggagtaaactcttcatacgttctctatcactgatagggagtaaactcgaatgaagacgaaATGCATGCGTCAATTTTACGCAGACTATCTTTCTAGGGTTAATCTAGCTGCATCAGGATCATATCGTCGGGTCTTTTTTCCGGCTCAGTCATCGCCCAAGCTGGCGCTATCTGGGCATCGGGGAGGAAGAAGCCCGTGCCTTTTCCCGCGAGGTTGAAGCGGCATGGAAAGAGTTTGCCGAGGATGACTGCTGCTGCATTGACGTTGAGCGAAAACGCACGTTTACCATGATGATTCGGGAAGGTGTGGCCATGCACGCCTTTAACGGTGAACTGTTCGTTCAGGCCACCTGGGATACCAGTTCGTCGCGGCTTTTCCGGACACAGTTCCGGATGGTCAGCCCGAAGCGCATCAGCAACCCGAACAATACCGGCGACAGCCGGAACTGCCGTGCCGGTGTGCAGATTAATGACAGCGGTGCGGCGCTGGGATATTACGTCAGCGAGGACGGGTATCCTGGCTGGATGCCGCAGAAATGGACATGGATACCCCGTGAGTTACCCGGCGGGCGCGCTTGGCGTAATCATGGTCATAGCTGTTTCCTGTGTGAAATTGTTATCCGCTCACAATTCCACACAACATACGAGCCGGAAGCATAAAGTGTAAAGCCTGGGGTGCCTAATGAGTGAGCTAACTCACATTAATTGCGTTGCGCTCACTGCCCGCTTTCCAGTCGGGAAACCTGTCGTGCCAGCTGCATTAATGAATCGGCCAACGCGCGGGGAGAGGCGGTTTGCGTATTGGGCGCTCTTCCGCTTCCTCGCTCACTGACTCGCTGCGCTCGGTCGTTCGGCTGCGGCGAGCGGTATCAGCTCACTCAAAGGCGGTAATACGGTTATCCACAGAATCAGGGGATAACGCAGGAAAGAACATGTGAGCAAAAGGCCAGCAAAAGGCCAGGAACCGTAAAAAGGCCGCGTTGCTGGCGTTTTTCCATAGGCTCCGCCCCCCTGACGAGCATCACAAAAATCGACGCTCAAGTCAGAGGTGGCGAAACCCGACAGGACTATAAAGATACCAGGCGTTTCCCCCTGGAAGCTCCCTCGTGCGCTCTCCTGTTCCGACCCTGCCGCTTACCGGATACCTGTCCGCCTTTCTCCCTTCGGGAAGCGTGGCGCTTTCTCATAGCTCACGCTGTAGGTATCTCAGTTCGGTGTAGGTCGTTCGCTCCAAGCTGGGCTGTGTGCACGAACCCCCCGTTCAGCCCGACCGCTGCGCCTTATCCGGTAACTATCGTCTTGAGTCCAACCCGGTAAGACACGACTTATCGCCACTGGCAGCAGCCACTGGTAACAGGATTAGCAGAGCGAGGTATGTAGGCGGTGCTACAGAGTTCTTGAAGTGGTGGCCTAACTACGGCTACACTAGAAGGACAGTATTTGGTATCTGCGCTCTGCTGAAGCCAGTTACCTTCGGAAAAAGAGTTGGTAGCTCTTGATCCGGCAAACAAACCACCGCTGGTAGCGGTGGTTTTTTTGTTTGCAAGCAGCAGATTACGCGCAGAAAAAAAGGATCTCAAGAAGATCCTTTGATCTTTTCTACGGGGTCTGACGCTCAGTGGAACGAAAACTCACGTTAAGGGATTTTGGTCATGAGATTATCAAAAAGGATCTTCACCTAGATCCTTTTAAATTAAAAATGAAGTTTTAAATCAATCTAAAGTATATATGAGTAAACTTGGTCTGACAGTTACCAATGCTTAATCAGTGAGGCACCTATCTCAGCGATCTGTCTATTTCGTTCATCCATAGTTGCCTGACTCCCCGTCGTGTAGATAACTACGATACGGGAGGGCTTACCATCTGGCCCCAGTGCTGCAATGATACCGCGAGACCCACGCTCACCGGCTCCAGATTTATCAGCAATAAACCAGCCAGCCGGAAGGGCCGAGCGCAGAAGTGGTCCTGCAACTTTATCCGCCTCCATCCAGTCTATTAATTGTTGCCGGGAAGCTAGAGTAAGTAGTTCGCCAGTTAATAGTTTGCGCAACGTTGTTGCCATTGCTACAGGCATCGTGGTGTCACGCTCGTCGTTTGGTATGGCTTCATTCAGCTCCGGTTCCCAACGATCAAGGCGAGTTACATGATCCCCCATGTTGTGCAAAAAAGCGGTTAGCTCCTTCGGTCCTCCGATCGTTGTCAGAAGTAAGTTGGCCGCAGTGTTATCACTCATGGTTATGGCAGCACTGCATAATTCTCTTACTGTCATGCCATCCGTAAGATGCTTTTCTGTGACTGGTGAGTACTCAACCAAGTCATTCTGAGAATAGTGTATGCGGCGACCGAGTTGCTCTTGCCCGGCGTCAATACGGGATAATACCGCGCCACATAGCAGAACTTTAAAAGTGCTCATCATTGGAAAACGTTCTTCGGGGCGAAAACTCTCAAGGATCTTACCGCTGTTGAGATCCAGTTCGATGTAACCCACTCGTGCACCCAACTGATCTTCAGCATCTTTTACTTTCACCAGCGTTTCTGGGTGAGCAAAAACAGGAAGGCAAAATGCCGCAAAAAAGGGAATAAGGGCGACACGGAAATGTTGAATACTCATACTCTTCCTTTTTCAATATTATTGAAGCATTTATCAGGGTTATTGTCTCATGAGCGGATACATATTTGAATGTATTTAGAAAAATAAACAAATAGGGGTTCCGCGCACATTTCCCCGAAAAGTGCCAC

plasmid 4

CTAAATTGTAAGCGTTAATATTTTGTTAAAATTCGCGTTAAATTTTTGTTAAATCAGCTCATTTTTTAACCAATAGGCCGAAATCGGCAAAATCCCTTATAAATCAAAAGAATAGACCGAGATAGGGTTGAGTGTTGTTCCAGTTTGGAACAAGAGTCCACTATTAAAGAACGTGGACTCCAACGTCAAAGGGCGAAAAACCGTCTATCAGGGCGATGGCCCACTACGTGAACCATCACCCTAATCAAGTTTTTTGGGGTCGAGGTGCCGTAAAGCACTAAATCGGAACCCTAAAGGGAGCCCCCGATTTAGAGCTTGACGGGGAAAGCCGGCGAACGTGGCGAGAAAGGAAGGGAAGAAAGCGAAAGGAGCGGGCGCTAGGGCGCTGGCAAGTGTAGCGGTCACGCTGCGCGTAACCACCACACCCGCCGCGCTTAATGCGCCGCTACAGGGCGCGTCCCATTCGCCATTCAGGCTGCGCAACTGTTGGGAAGGGCGATCGGTGCGGGCCTCTTCGCTATTACGCCAGCTGGCGAAAGGGGGATGTGCTGCAAGGCGATTAAGTTGGGTAACGCCAGGGTTTTCCCAGTCACGACGTTGTAAAACGACGGCCAGTGAGCGCGCCTCGTTCATTCACGTTTTTGAACCCGTGGAGGACGGGCAGACTCGCGGTGCAAATGTGTTTTACAGCGTGATGGAGCAGATGAAGATGCTCGACACGCTGCAGAACACGCAGCTAGATTAACCCTAGAAAGATAATCATATTGTGACGTACGTTAAAGATAATCATGCGTAAAATTGACGCATGTGTTTTATCGGTCTGTATATCGAGGTTTATTTATTAATTTGAATAGATATTAAGTTTTATTATATTTACACTTACATACTAATAATAAATTCAACAAACAATTTATTTATGTTTATTTATTTATTAAAAAAAAACAAAAACTCAAAATTTCTTCTATAAAGTAACAAAACTTTTATCGAATTCGATGTCTTTGTGATGCGCGCGACATTTTTGTAGGTTATTGATAAAATGAACGGATACGTTGCCCGACATTATCATTAAATCCTTGGCGTAGAATTTGTCGGGTCCATTGTCCGTGTGCGCTAGCATGCCCGTAACGGACCTCGTACTTTTGGCTTCAAAGGTTTTGCGCACAGACAAAATGTGCCACACTTGCAGCTCTGCATGTGTGCGCGTTACCACAAATCCCAACGGCGCAGTGTACTTGTTGTATGCAAATAAATCTCGATAAAGGCGCGGCGCGCGAATGCAGCTGATCACGTACGCTCCTCGTGTTCCGTTCAAGGACGGTGTTATCGACCTCAGATTAATGTTTATCGGCCGACTGTTTTCGTATCCGCTCACCAAACGCGTTTTTGCATTAACATTGTATGTCGGCGGATGTTCTATATCTAATTTGAATAAATAAACGATAACCGCGTTGGTTTTAGAGGGCATAATAAAAGAAATATTGTTATCGTGTTCGCCATTAGGGCAGTATAAATTGACGTTCATGTTGGATATTGTTTCAGTTGCAAGTTGACACTGGCGGCGACAAGATCGTGAACAACCAAGTGACTATTACGCAAATTAATTTTAACGCGccaccatgtcCagattagataaaagtaaagtgattaacagcgcattagagctgcttaatgaggtcggaatcgaaggtttaacaacccgtaaactcgcccagaagctaggtgtagagcagcctacattgtattggcacgtgcgcaacaagcagactcttatgaacatgctttcagaggcaatactggcgaagcatcacacccgttcagcaccgttaccgactgagagttggcagcagtttctccaggaaaatgctctgagtttccgtaaagcattactggtccatcgtgatggagcccgattgcatatagggacctctcctacgcccccccagtttgaacaagcagaggcgcaactacgctgtctatgcgatgcagggttttcggtcgaggaggctcttttcattctgcaatctatcagccattttacgttgggtgcagtattagaggagcaagcaacaaaccagatagaaaataatcatgtgatagacgctgcaccaccattattacaagaggcatttaatattcaggcgagaacctctgctgaaatggccttccatttcgggctgaaatcattaatatttggattttctgcacagttagatgaaaaaaagcatacacccattgaggatggtaataaaccaaaaaagaagagaaagctagcagtgtcagtgacatttgaagatgtggctgtgctctttactcgggacgagtggaagaagctggatctgtctcagagaagcctgtaccgtgaggtgatgctggagaattacagcaacctggcctccatggcaggattcctgtttaccaaaccaaaggtgatctccctgttgcagcaaggagaggaCccctggtaaGGTACCGGGAGATGGGGGAGGCTAACTGAAACACGGAAGGAGACAATACCGGAAGGAACCCGCGCTATGACGGCAATAAAAAGACAGAATAAAACGCACGGGTGTTGGGTCGTTTGTTCactagtatcaagtgcggaaatttattaaaacacaatttatttcacaatacaattttattaagtaaagttataatgcaagtgtatatagggctacaatatagtatacatggattacaatgtgggtatacaaaagctatagtatggatataaaattattaagcacattggggttacaatataaacataatcaaccaCTTAAGtcacttgtacagctcgtccatgccgccggtggagtggcggccctcggcgcgttcgtactgttccacgatggtgtagtcctcgttgtgggaggtgatgtccaacttgatgttgacgttgtaggcgccgggcagctgcacgggcttcttggccttgtaggtggtcttgacctcagcgtcgtagtggccgccgtccttcagcttcagcctctgcttgatctcgcccttcagggcgccgtcctcggggtacatccgctcggaggaggcctcccagcccatggtTttcttctgcattacggggccgtcggaggggaagttggtgccgcgcagcttcaccttgtagatgaactcgccgtcctgcagggaggagtcctgggtcacggtcaccacgccgccgtcctcgaagttcatcacgcgctcccacttgaagccctcggggaaggacagcttcaagtagtcggggatgtcggcggggtgcttcacgtaggccttggagccgtacatgaactgaggggacaggatgtcccaggcgaagggcagggggccacccttggtcaccttcagcttggcggtctgggtgccctcgtaggggcggccctcgccctcgccctcgatctcgaactcgtggccgttcacggagccctccatgtgcaccttgaagcgcatgaactccttgatgatggccatgttatcctcctcgcccttgctcaccatggtggcaccggtcttgagtggagagagagagctagttataagcgtgtgtgtgcgctcttgtgactgaatcgaggagctaacacgggctcttatatacacaaatatgctccgcccacaacagcgattacaccatttctaggaataaatgagtaacaaggGGATCCcatgatgataaacaatgtatggtgctaatgttgcttcaacaacaattctgttgaactgtgttttcatgtttgccaacaagcacctttatactcggtggcctccccaccaccaacttttttgcactgcaaaaaaacacgcttttgcacgcgggcccatacatagtacaaactctacgtttcgtagactattttacataaatagtctacaccgttgtatacgctccaaatacactaccacacattgaacctttttgcagtgcaaaaaagtacgtgtcggcagtcacgtaggccggccttatcgggtcgcgtcctgtcacgtacgaatcacattatcggaccggacgagtgttgtcttatcgtgacaggacgccagcttcctgtgttgctaaccgcagccggacgcaactccttatcggaacaggacgcgcctccatatcagccgcgcgttatctcatgcgcgtgaccggacacgaggcgcccgtcccgcttatcgcgcctataaatacagcccgcaacgatctggtaaacacagttgaacagcatctgttacagcgacacaacGGTACCgccACCatgtctagactggacaagagcaaagtcataaactctgctctggaattactcaatggagtcggtatcgaaggcctgacgacaaggaaactcgctcaaaagctgggagttgagcagcctaccctgtactggcacgtgaagaacaagcgggccctgctcgatgccctgccaatcgagatgctggacaggcatcatacccactcctgccccctggaaggcgagtcatggcaagactttctgcggaacaacgccaagtcataccgctgtgctctcctctcacatcgcgacggggctaaagtgcatctcggcacccgcccaacagagaaacagtacgaaaccctggaaaatcagctcgcgttcctgtgtcagcaaggcttctccctggagaacgcactgtacgctctgtccgccgtgggccactttacactgggctgcgtattggaggaacaggagcatcaagtagcaaaagaggaaagagagacacctaccaccgattctatgcccccacttctgaaacaagcaattgagctgttcgaccggcagggagccgaacctgccttccttttcggcctggaactaatcatatgtggcctggagaaacagctaaagtgcgaaagcggcgggccgaccgacgcccttgacgattttgacttagacatgctcccagccgatgcccttgacgactttgaccttgatatgctgcctgctgacgctcttgacgattttgaccttgacatgctccccgggtaaGGCGCGCCtttgtattgtcatgttttaatacaatatgttatgtttaaatatgtttttaataaattttataaaataatttcaacttttattgtaacaacattgtccatttacacactcctttcaagcgcgtgcgcacccgaaaagcagggtcgccgctgacgcactgctaaaaatagcacgcgcctttcaaaagcgttcacacccgaaaCTCGAGGTAAGATACATTGATGAGTTTGGACAAACCACAACTAGAATGCAGTGAAAAAAATGCTTTATTTGTGAAATTTGTGATGCTATTGCTTTATTTGTAACCATTATAAGCTGCAATAAACAAGTTAACAACAACAATTGCATTCATTTTATGTTTCAGGTTCAGGGGGAGGTGTGGGAGGTTTTTTAAAGCAAGTAAAACCTCTACAAATGTGGTATGGCTGATTATGATCGTCGACtcaagtcagaatagtggacaggcggccaagaacttcgttcatgatagtctccggaacccgttcgagtcgttttccgccccgtgctttcatatcaattgtccggggttgatcgcaacgtacaacacctgtggtacgtatgccaacaccatccaacgacaccgcaaagccggcagtgcgggcaaaattgcctccgctggttacgggcacaacaacaggcaggcgggtcacgcgattaaaggccgccggtgtgacaatcagcaccggccgcgttccctgctgctcatgacctgcggtaggatcaagcgagacaagccagatttcccctctttccatggtgGCGGCCGCaagtataagacaaaagtgttgtggaattgctccaggcgatctgacggttcactaaacgagctctgcttttataggcgccCACCGTACACGCCTAAAGCTTATACGTtctctatcactgatagggagtaaactggatatacgttctctatcactgatagggagtaaactgtagatacgttctctatcactgatagggagtaaactggtcatacgttctctatcactgatagggagtaaactccttatacgttctctatcactgatagggagtaaagtctgcatacgttctctatcactgatagggagtaaactcttcatacgttctctatcactgatagggagtaaactcgaatgaagacgaaATGCATGCGTCAATTTTACGCAGACTATCTTTCTAGGGTTAATCTAGCTGCATCAGGATCATATCGTCGGGTCTTTTTTCCGGCTCAGTCATCGCCCAAGCTGGCGCTATCTGGGCATCGGGGAGGAAGAAGCCCGTGCCTTTTCCCGCGAGGTTGAAGCGGCATGGAAAGAGTTTGCCGAGGATGACTGCTGCTGCATTGACGTTGAGCGAAAACGCACGTTTACCATGATGATTCGGGAAGGTGTGGCCATGCACGCCTTTAACGGTGAACTGTTCGTTCAGGCCACCTGGGATACCAGTTCGTCGCGGCTTTTCCGGACACAGTTCCGGATGGTCAGCCCGAAGCGCATCAGCAACCCGAACAATACCGGCGACAGCCGGAACTGCCGTGCCGGTGTGCAGATTAATGACAGCGGTGCGGCGCTGGGATATTACGTCAGCGAGGACGGGTATCCTGGCTGGATGCCGCAGAAATGGACATGGATACCCCGTGAGTTACCCGGCGGGCGCGCTTGGCGTAATCATGGTCATAGCTGTTTCCTGTGTGAAATTGTTATCCGCTCACAATTCCACACAACATACGAGCCGGAAGCATAAAGTGTAAAGCCTGGGGTGCCTAATGAGTGAGCTAACTCACATTAATTGCGTTGCGCTCACTGCCCGCTTTCCAGTCGGGAAACCTGTCGTGCCAGCTGCATTAATGAATCGGCCAACGCGCGGGGAGAGGCGGTTTGCGTATTGGGCGCTCTTCCGCTTCCTCGCTCACTGACTCGCTGCGCTCGGTCGTTCGGCTGCGGCGAGCGGTATCAGCTCACTCAAAGGCGGTAATACGGTTATCCACAGAATCAGGGGATAACGCAGGAAAGAACATGTGAGCAAAAGGCCAGCAAAAGGCCAGGAACCGTAAAAAGGCCGCGTTGCTGGCGTTTTTCCATAGGCTCCGCCCCCCTGACGAGCATCACAAAAATCGACGCTCAAGTCAGAGGTGGCGAAACCCGACAGGACTATAAAGATACCAGGCGTTTCCCCCTGGAAGCTCCCTCGTGCGCTCTCCTGTTCCGACCCTGCCGCTTACCGGATACCTGTCCGCCTTTCTCCCTTCGGGAAGCGTGGCGCTTTCTCATAGCTCACGCTGTAGGTATCTCAGTTCGGTGTAGGTCGTTCGCTCCAAGCTGGGCTGTGTGCACGAACCCCCCGTTCAGCCCGACCGCTGCGCCTTATCCGGTAACTATCGTCTTGAGTCCAACCCGGTAAGACACGACTTATCGCCACTGGCAGCAGCCACTGGTAACAGGATTAGCAGAGCGAGGTATGTAGGCGGTGCTACAGAGTTCTTGAAGTGGTGGCCTAACTACGGCTACACTAGAAGGACAGTATTTGGTATCTGCGCTCTGCTGAAGCCAGTTACCTTCGGAAAAAGAGTTGGTAGCTCTTGATCCGGCAAACAAACCACCGCTGGTAGCGGTGGTTTTTTTGTTTGCAAGCAGCAGATTACGCGCAGAAAAAAAGGATCTCAAGAAGATCCTTTGATCTTTTCTACGGGGTCTGACGCTCAGTGGAACGAAAACTCACGTTAAGGGATTTTGGTCATGAGATTATCAAAAAGGATCTTCACCTAGATCCTTTTAAATTAAAAATGAAGTTTTAAATCAATCTAAAGTATATATGAGTAAACTTGGTCTGACAGTTACCAATGCTTAATCAGTGAGGCACCTATCTCAGCGATCTGTCTATTTCGTTCATCCATAGTTGCCTGACTCCCCGTCGTGTAGATAACTACGATACGGGAGGGCTTACCATCTGGCCCCAGTGCTGCAATGATACCGCGAGACCCACGCTCACCGGCTCCAGATTTATCAGCAATAAACCAGCCAGCCGGAAGGGCCGAGCGCAGAAGTGGTCCTGCAACTTTATCCGCCTCCATCCAGTCTATTAATTGTTGCCGGGAAGCTAGAGTAAGTAGTTCGCCAGTTAATAGTTTGCGCAACGTTGTTGCCATTGCTACAGGCATCGTGGTGTCACGCTCGTCGTTTGGTATGGCTTCATTCAGCTCCGGTTCCCAACGATCAAGGCGAGTTACATGATCCCCCATGTTGTGCAAAAAAGCGGTTAGCTCCTTCGGTCCTCCGATCGTTGTCAGAAGTAAGTTGGCCGCAGTGTTATCACTCATGGTTATGGCAGCACTGCATAATTCTCTTACTGTCATGCCATCCGTAAGATGCTTTTCTGTGACTGGTGAGTACTCAACCAAGTCATTCTGAGAATAGTGTATGCGGCGACCGAGTTGCTCTTGCCCGGCGTCAATACGGGATAATACCGCGCCACATAGCAGAACTTTAAAAGTGCTCATCATTGGAAAACGTTCTTCGGGGCGAAAACTCTCAAGGATCTTACCGCTGTTGAGATCCAGTTCGATGTAACCCACTCGTGCACCCAACTGATCTTCAGCATCTTTTACTTTCACCAGCGTTTCTGGGTGAGCAAAAACAGGAAGGCAAAATGCCGCAAAAAAGGGAATAAGGGCGACACGGAAATGTTGAATACTCATACTCTTCCTTTTTCAATATTATTGAAGCATTTATCAGGGTTATTGTCTCATGAGCGGATACATATTTGAATGTATTTAGAAAAATAAACAAATAGGGGTTCCGCGCACATTTCCCCGAAAAGTGCCAC

plasmid 5

CTAAATTGTAAGCGTTAATATTTTGTTAAAATTCGCGTTAAATTTTTGTTAAATCAGCTCATTTTTTAACCAATAGGCCGAAATCGGCAAAATCCCTTATAAATCAAAAGAATAGACCGAGATAGGGTTGAGTGTTGTTCCAGTTTGGAACAAGAGTCCACTATTAAAGAACGTGGACTCCAACGTCAAAGGGCGAAAAACCGTCTATCAGGGCGATGGCCCACTACGTGAACCATCACCCTAATCAAGTTTTTTGGGGTCGAGGTGCCGTAAAGCACTAAATCGGAACCCTAAAGGGAGCCCCCGATTTAGAGCTTGACGGGGAAAGCCGGCGAACGTGGCGAGAAAGGAAGGGAAGAAAGCGAAAGGAGCGGGCGCTAGGGCGCTGGCAAGTGTAGCGGTCACGCTGCGCGTAACCACCACACCCGCCGCGCTTAATGCGCCGCTACAGGGCGCGTCCCATTCGCCATTCAGGCTGCGCAACTGTTGGGAAGGGCGATCGGTGCGGGCCTCTTCGCTATTACGCCAGCTGGCGAAAGGGGGATGTGCTGCAAGGCGATTAAGTTGGGTAACGCCAGGGTTTTCCCAGTCACGACGTTGTAAAACGACGGCCAGTGAGCGCGCCTCGTTCATTCACGTTTTTGAACCCGTGGAGGACGGGCAGACTCGCGGTGCAAATGTGTTTTACAGCGTGATGGAGCAGATGAAGATGCTCGACACGCTGCAGAACACGCAGCTAGATTAACCCTAGAAAGATAATCATATTGTGACGTACGTTAAAGATAATCATGCGTAAAATTGACGCATGTGTTTTATCGGTCTGTATATCGAGGTTTATTTATTAATTTGAATAGATATTAAGTTTTATTATATTTACACTTACATACTAATAATAAATTCAACAAACAATTTATTTATGTTTATTTATTTATTAAAAAAAAACAAAAACTCAAAATTTCTTCTATAAAGTAACAAAACTTTTATCGAATTCatacgaaatgaactagtatcaagtgcggaaatttattaaaacacaatttatttcacaatacaattttattaagtaaagttataatgcaagtgtatatagggctacaatatagtatacatggattacaatgtgggtatacaaaagctatagtatggatataaaattattaagcacattggggttacaatataaacataatcaaccaCTTAAGtcacttgtacagctcgtccatgccgccggtggagtggcggccctcggcgcgttcgtactgttccacgatggtgtagtcctcgttgtgggaggtgatgtccaacttgatgttgacgttgtaggcgccgggcagctgcacgggcttcttggccttgtaggtggtcttgacctcagcgtcgtagtggccgccgtccttcagcttcagcctctgcttgatctcgcccttcagggcgccgtcctcggggtacatccgctcggaggaggcctcccagcccatggtTttcttctgcattacggggccgtcggaggggaagttggtgccgcgcagcttcaccttgtagatgaactcgccgtcctgcagggaggagtcctgggtcacggtcaccacgccgccgtcctcgaagttcatcacgcgctcccacttgaagccctcggggaaggacagcttcaagtagtcggggatgtcggcggggtgcttcacgtaggccttggagccgtacatgaactgaggggacaggatgtcccaggcgaagggcagggggccacccttggtcaccttcagcttggcggtctgggtgccctcgtaggggcggccctcgccctcgccctcgatctcgaactcgtggccgttcacggagccctccatgtgcaccttgaagcgcatgaactccttgatgatggccatgttatcctcctcgcccttgctcaccatggtggcaccggtcttgagtggagagagagagctagttataagcgtgtgtgtgcgctcttgtgactgaatcgaggagctaacacgggctcttatatacacaaatatgctccgcccacaacagcgattacaccatttctaggaataaatgagtaacaaggGGATCCcatgatgataaacaatgtatggtgctaatgttgcttcaacaacaattctgttgaactgtgttttcatgtttgccaacaagcacctttatactcggtggcctccccaccaccaacttttttgcactgcaaaaaaacacgcttttgcacgcgggcccatacatagtacaaactctacgtttcgtagactattttacataaatagtctacaccgttgtatacgctccaaatacactaccacacattgaacctttttgcagtgcaaaaaagtacgtgtcggcagtcacgtaggccggccttatcgggtcgcgtcctgtcacgtacgaatcacattatcggaccggacgagtgttgtcttatcgtgacaggacgccagcttcctgtgttgctaaccgcagccggacgcaactccttatcggaacaggacgcgcctccatatcagccgcgcgttatctcatgcgcgtgaccggacacgaggcgcccgtcccgcttatcgcgcctataaatacagcccgcaacgatctggtaaacacagttgaacagcatctgttacagcgacacaacGGTACCgccACCatgtctagactggacaagagcaaagtcataaactctgctctggaattactcaatggagtcggtatcgaaggcctgacgacaaggaaactcgctcaaaagctgggagttgagcagcctaccctgtactggcacgtgaagaacaagcgggccctgctcgatgccctgccaatcgagatgctggacaggcatcatacccactcctgccccctggaaggcgagtcatggcaagactttctgcggaacaacgccaagtcataccgctgtgctctcctctcacatcgcgacggggctaaagtgcatctcggcacccgcccaacagagaaacagtacgaaaccctggaaaatcagctcgcgttcctgtgtcagcaaggcttctccctggagaacgcactgtacgctctgtccgccgtgggccactttacactgggctgcgtattggaggaacaggagcatcaagtagcaaaagaggaaagagagacacctaccaccgattctatgcccccacttctgaaacaagcaattgagctgttcgaccggcagggagccgaacctgccttccttttcggcctggaactaatcatatgtggcctggagaaacagctaaagtgcgaaagcggcgggccgaccgacgcccttgacgattttgacttagacatgctcccagccgatgcccttgacgactttgaccttgatatgctgcctgctgacgctcttgacgattttgaccttgacatgctccccgggtaaGGCGCGCCtttgtattgtcatgttttaatacaatatgttatgtttaaatatgtttttaataaattttataaaataatttcaacttttattgtaacaacattgtccatttacacactcctttcaagcgcgtgcgcacccgaaaagcagggtcgccgctgacgcactgctaaaaatagcacgcgcctttcaaaagcgttcacacccgaaaCTCGAGGTAAGATACATTGATGAGTTTGGACAAACCACAACTAGAATGCAGTGAAAAAAATGCTTTATTTGTGAAATTTGTGATGCTATTGCTTTATTTGTAACCATTATAAGCTGCAATAAACAAGTTAACAACAACAATTGCATTCATTTTATGTTTCAGGTTCAGGGGGAGGTGTGGGAGGTTTTTTAAAGCAAGTAAAACCTCTACAAATGTGGTATGGCTGATTATGATCGTCGACttacaatttggactttccgcccttcttggcctttatgaggatctctctgatttttcttgcgtcgagttttccggtaagacctttcggtacttcgtccacaaacacaactcctccgcgcaactttttggcggttgttacttgactggcgacgtaatccacgatctctttttccgtcatcgtctttccgtgctccaaaacaacaacggcggcgggaagttcaccggcgtcatcgtcgggaagacctgccacgcccgcgtcgaagatgttggggtgttgtaacaatatcgactccaattcagcgggggccacctggtatcctttgtatttaattaaagacttcaagcggtcaactatgaagaagtgttcgtcttcgtcccagtaagctatgtctccagaatgtagccatccatccttgtcaatcaaggcgttggtcgcttccggattgtttacataaccggacataatcataggtcctctgacacataattcgcctctctgattaacgcccagcgttttcccggtatccagatccacaaccttcgcttcaaaaaatggaacaactttaccgaccgcgcccggtttatcatccccctcgggtgtaatcagaatagctgatgtagtctcagtgagcccatatccttgtcgtatccctggaagatggaagcgttttgcaaccgcttccccgacttctttcgaaagaggtgcgcccccagaagcaatttcgtgtaaattagataaatcgtatttgtcaatcagagtgcttttggcgaagaatgaaaatagggttggtactagcaacgcactttgaattttgtaatcctgaagggatcgtaaaaacagctcttcttcaaatctatacattaagacgactcgaaatccacatatcaaatatccgagtgtagtaaacattccaaaaccgtgatggaatggaacaacacttaaaatcgcagtatccggaatgatttgattgccaaaaataggatctctggcatgcgagaatctgacgcaggcagttctatgcggaagggccacacccttaggtaacccagtagatccagaggagttcattatcagtgcaattgttttgtcacgatcaaaggactctggtacaaaatcgtattcattaaaaccgggaggtagatgagatgtgacgaacgtgtacatcgactgaaatccctggtaatccgttttagaatccatgataataattttctggattattggtaattttttttgcacgttcaaaattttttgcaacccctttttggaaacaaacactacggtaggctgcgaaatgttcatactgttgagcaattcacgttcattataaatgtcgttcgcgggcgcaactgcaactccgataaataacgcgcccaacaccggcataaagaattgaagagagttttcactgcatacgacgattctgtgatttgtattcagcccatatcgtttcatagcttctgccaaccgaacggacatttcgaagtattccgcgtacgtgatgttcacctcgatatgtgcatctgtaaaagcaattgttccaggaaccagggcgtatctcttcatagccttatgcagttgctctccagcggttccatcctctagcggatagaatggcgccgggcctttctttatgtttttggcgtcttccatggtgGCGGCCGCaagtataagacaaaagtgttgtggaattgctccaggcgatctgacggttcactaaacgagctctgcttttataggcgccCACCGTACACGCCTAAAGCTTATACGTtctctatcactgatagggagtaaactggatatacgttctctatcactgatagggagtaaactgtagatacgttctctatcactgatagggagtaaactggtcatacgttctctatcactgatagggagtaaactccttatacgttctctatcactgatagggagtaaagtctgcatacgttctctatcactgatagggagtaaactcttcatacgttctctatcactgatagggagtaaactcgaatgaagacgaaATGCATGCGTCAATTTTACGCAGACTATCTTTCTAGGGTTAATCTAGCTGCATCAGGATCATATCGTCGGGTCTTTTTTCCGGCTCAGTCATCGCCCAAGCTGGCGCTATCTGGGCATCGGGGAGGAAGAAGCCCGTGCCTTTTCCCGCGAGGTTGAAGCGGCATGGAAAGAGTTTGCCGAGGATGACTGCTGCTGCATTGACGTTGAGCGAAAACGCACGTTTACCATGATGATTCGGGAAGGTGTGGCCATGCACGCCTTTAACGGTGAACTGTTCGTTCAGGCCACCTGGGATACCAGTTCGTCGCGGCTTTTCCGGACACAGTTCCGGATGGTCAGCCCGAAGCGCATCAGCAACCCGAACAATACCGGCGACAGCCGGAACTGCCGTGCCGGTGTGCAGATTAATGACAGCGGTGCGGCGCTGGGATATTACGTCAGCGAGGACGGGTATCCTGGCTGGATGCCGCAGAAATGGACATGGATACCCCGTGAGTTACCCGGCGGGCGCGCTTGGCGTAATCATGGTCATAGCTGTTTCCTGTGTGAAATTGTTATCCGCTCACAATTCCACACAACATACGAGCCGGAAGCATAAAGTGTAAAGCCTGGGGTGCCTAATGAGTGAGCTAACTCACATTAATTGCGTTGCGCTCACTGCCCGCTTTCCAGTCGGGAAACCTGTCGTGCCAGCTGCATTAATGAATCGGCCAACGCGCGGGGAGAGGCGGTTTGCGTATTGGGCGCTCTTCCGCTTCCTCGCTCACTGACTCGCTGCGCTCGGTCGTTCGGCTGCGGCGAGCGGTATCAGCTCACTCAAAGGCGGTAATACGGTTATCCACAGAATCAGGGGATAACGCAGGAAAGAACATGTGAGCAAAAGGCCAGCAAAAGGCCAGGAACCGTAAAAAGGCCGCGTTGCTGGCGTTTTTCCATAGGCTCCGCCCCCCTGACGAGCATCACAAAAATCGACGCTCAAGTCAGAGGTGGCGAAACCCGACAGGACTATAAAGATACCAGGCGTTTCCCCCTGGAAGCTCCCTCGTGCGCTCTCCTGTTCCGACCCTGCCGCTTACCGGATACCTGTCCGCCTTTCTCCCTTCGGGAAGCGTGGCGCTTTCTCATAGCTCACGCTGTAGGTATCTCAGTTCGGTGTAGGTCGTTCGCTCCAAGCTGGGCTGTGTGCACGAACCCCCCGTTCAGCCCGACCGCTGCGCCTTATCCGGTAACTATCGTCTTGAGTCCAACCCGGTAAGACACGACTTATCGCCACTGGCAGCAGCCACTGGTAACAGGATTAGCAGAGCGAGGTATGTAGGCGGTGCTACAGAGTTCTTGAAGTGGTGGCCTAACTACGGCTACACTAGAAGGACAGTATTTGGTATCTGCGCTCTGCTGAAGCCAGTTACCTTCGGAAAAAGAGTTGGTAGCTCTTGATCCGGCAAACAAACCACCGCTGGTAGCGGTGGTTTTTTTGTTTGCAAGCAGCAGATTACGCGCAGAAAAAAAGGATCTCAAGAAGATCCTTTGATCTTTTCTACGGGGTCTGACGCTCAGTGGAACGAAAACTCACGTTAAGGGATTTTGGTCATGAGATTATCAAAAAGGATCTTCACCTAGATCCTTTTAAATTAAAAATGAAGTTTTAAATCAATCTAAAGTATATATGAGTAAACTTGGTCTGACAGTTACCAATGCTTAATCAGTGAGGCACCTATCTCAGCGATCTGTCTATTTCGTTCATCCATAGTTGCCTGACTCCCCGTCGTGTAGATAACTACGATACGGGAGGGCTTACCATCTGGCCCCAGTGCTGCAATGATACCGCGAGACCCACGCTCACCGGCTCCAGATTTATCAGCAATAAACCAGCCAGCCGGAAGGGCCGAGCGCAGAAGTGGTCCTGCAACTTTATCCGCCTCCATCCAGTCTATTAATTGTTGCCGGGAAGCTAGAGTAAGTAGTTCGCCAGTTAATAGTTTGCGCAACGTTGTTGCCATTGCTACAGGCATCGTGGTGTCACGCTCGTCGTTTGGTATGGCTTCATTCAGCTCCGGTTCCCAACGATCAAGGCGAGTTACATGATCCCCCATGTTGTGCAAAAAAGCGGTTAGCTCCTTCGGTCCTCCGATCGTTGTCAGAAGTAAGTTGGCCGCAGTGTTATCACTCATGGTTATGGCAGCACTGCATAATTCTCTTACTGTCATGCCATCCGTAAGATGCTTTTCTGTGACTGGTGAGTACTCAACCAAGTCATTCTGAGAATAGTGTATGCGGCGACCGAGTTGCTCTTGCCCGGCGTCAATACGGGATAATACCGCGCCACATAGCAGAACTTTAAAAGTGCTCATCATTGGAAAACGTTCTTCGGGGCGAAAACTCTCAAGGATCTTACCGCTGTTGAGATCCAGTTCGATGTAACCCACTCGTGCACCCAACTGATCTTCAGCATCTTTTACTTTCACCAGCGTTTCTGGGTGAGCAAAAACAGGAAGGCAAAATGCCGCAAAAAAGGGAATAAGGGCGACACGGAAATGTTGAATACTCATACTCTTCCTTTTTCAATATTATTGAAGCATTTATCAGGGTTATTGTCTCATGAGCGGATACATATTTGAATGTATTTAGAAAAATAAACAAATAGGGGTTCCGCGCACATTTCCCCGAAAAGTGCCAC

plasmid 6

CTAAATTGTAAGCGTTAATATTTTGTTAAAATTCGCGTTAAATTTTTGTTAAATCAGCTCATTTTTTAACCAATAGGCCGAAATCGGCAAAATCCCTTATAAATCAAAAGAATAGACCGAGATAGGGTTGAGTGTTGTTCCAGTTTGGAACAAGAGTCCACTATTAAAGAACGTGGACTCCAACGTCAAAGGGCGAAAAACCGTCTATCAGGGCGATGGCCCACTACGTGAACCATCACCCTAATCAAGTTTTTTGGGGTCGAGGTGCCGTAAAGCACTAAATCGGAACCCTAAAGGGAGCCCCCGATTTAGAGCTTGACGGGGAAAGCCGGCGAACGTGGCGAGAAAGGAAGGGAAGAAAGCGAAAGGAGCGGGCGCTAGGGCGCTGGCAAGTGTAGCGGTCACGCTGCGCGTAACCACCACACCCGCCGCGCTTAATGCGCCGCTACAGGGCGCGTCCCATTCGCCATTCAGGCTGCGCAACTGTTGGGAAGGGCGATCGGTGCGGGCCTCTTCGCTATTACGCCAGCTGGCGAAAGGGGGATGTGCTGCAAGGCGATTAAGTTGGGTAACGCCAGGGTTTTCCCAGTCACGACGTTGTAAAACGACGGCCAGTGAGCGCGCCTCGTTCATTCACGTTTTTGAACCCGTGGAGGACGGGCAGACTCGCGGTGCAAATGTGTTTTACAGCGTGATGGAGCAGATGAAGATGCTCGACACGCTGCAGAACACGCAGCTAGATTAACCCTAGAAAGATAATCATATTGTGACGTACGTTAAAGATAATCATGCGTAAAATTGACGCATGTGTTTTATCGGTCTGTATATCGAGGTTTATTTATTAATTTGAATAGATATTAAGTTTTATTATATTTACACTTACATACTAATAATAAATTCAACAAACAATTTATTTATGTTTATTTATTTATTAAAAAAAAACAAAAACTCAAAATTTCTTCTATAAAGTAACAAAACTTTTATCGAATTCGATGTCTTTGTGATGCGCGCGACATTTTTGTAGGTTATTGATAAAATGAACGGATACGTTGCCCGACATTATCATTAAATCCTTGGCGTAGAATTTGTCGGGTCCATTGTCCGTGTGCGCTAGCATGCCCGTAACGGACCTCGTACTTTTGGCTTCAAAGGTTTTGCGCACAGACAAAATGTGCCACACTTGCAGCTCTGCATGTGTGCGCGTTACCACAAATCCCAACGGCGCAGTGTACTTGTTGTATGCAAATAAATCTCGATAAAGGCGCGGCGCGCGAATGCAGCTGATCACGTACGCTCCTCGTGTTCCGTTCAAGGACGGTGTTATCGACCTCAGATTAATGTTTATCGGCCGACTGTTTTCGTATCCGCTCACCAAACGCGTTTTTGCATTAACATTGTATGTCGGCGGATGTTCTATATCTAATTTGAATAAATAAACGATAACCGCGTTGGTTTTAGAGGGCATAATAAAAGAAATATTGTTATCGTGTTCGCCATTAGGGCAGTATAAATTGACGTTCATGTTGGATATTGTTTCAGTTGCAAGTTGACACTGGCGGCGACAAGATCGTGAACAACCAAGTGACTATTACGCAAATTAATTTTAACGCGccaccatgtcCagattagataaaagtaaagtgattaacagcgcattagagctgcttaatgaggtcggaatcgaaggtttaacaacccgtaaactcgcccagaagctaggtgtagagcagcctacattgtattggcacgtgcgcaacaagcagactcttatgaacatgctttcagaggcaatactggcgaagcatcacacccgttcagcaccgttaccgactgagagttggcagcagtttctccaggaaaatgctctgagtttccgtaaagcattactggtccatcgtgatggagcccgattgcatatagggacctctcctacgcccccccagtttgaacaagcagaggcgcaactacgctgtctatgcgatgcagggttttcggtcgaggaggctcttttcattctgcaatctatcagccattttacgttgggtgcagtattagaggagcaagcaacaaaccagatagaaaataatcatgtgatagacgctgcaccaccattattacaagaggcatttaatattcaggcgagaacctctgctgaaatggccttccatttcgggctgaaatcattaatatttggattttctgcacagttagatgaaaaaaagcatacacccattgaggatggtaataaaccaaaaaagaagagaaagctagcagtgtcagtgacatttgaagatgtggctgtgctctttactcgggacgagtggaagaagctggatctgtctcagagaagcctgtaccgtgaggtgatgctggagaattacagcaacctggcctccatggcaggattcctgtttaccaaaccaaaggtgatctccctgttgcagcaaggagaggaCccctggtaaGGTACCGGGAGATGGGGGAGGCTAACTGAAACACGGAAGGAGACAATACCGGAAGGAACCCGCGCTATGACGGCAATAAAAAGACAGAATAAAACGCACGGGTGTTGGGTCGTTTGTTCactagtatcaagtgcggaaatttattaaaacacaatttatttcacaatacaattttattaagtaaagttataatgcaagtgtatatagggctacaatatagtatacatggattacaatgtgggtatacaaaagctatagtatggatataaaattattaagcacattggggttacaatataaacataatcaaccaCTTAAGtcacttgtacagctcgtccatgccgccggtggagtggcggccctcggcgcgttcgtactgttccacgatggtgtagtcctcgttgtgggaggtgatgtccaacttgatgttgacgttgtaggcgccgggcagctgcacgggcttcttggccttgtaggtggtcttgacctcagcgtcgtagtggccgccgtccttcagcttcagcctctgcttgatctcgcccttcagggcgccgtcctcggggtacatccgctcggaggaggcctcccagcccatggtTttcttctgcattacggggccgtcggaggggaagttggtgccgcgcagcttcaccttgtagatgaactcgccgtcctgcagggaggagtcctgggtcacggtcaccacgccgccgtcctcgaagttcatcacgcgctcccacttgaagccctcggggaaggacagcttcaagtagtcggggatgtcggcggggtgcttcacgtaggccttggagccgtacatgaactgaggggacaggatgtcccaggcgaagggcagggggccacccttggtcaccttcagcttggcggtctgggtgccctcgtaggggcggccctcgccctcgccctcgatctcgaactcgtggccgttcacggagccctccatgtgcaccttgaagcgcatgaactccttgatgatggccatgttatcctcctcgcccttgctcaccatggtggcaccggtcttgagtggagagagagagctagttataagcgtgtgtgtgcgctcttgtgactgaatcgaggagctaacacgggctcttatatacacaaatatgctccgcccacaacagcgattacaccatttctaggaataaatgagtaacaaggGGATCCcatgatgataaacaatgtatggtgctaatgttgcttcaacaacaattctgttgaactgtgttttcatgtttgccaacaagcacctttatactcggtggcctccccaccaccaacttttttgcactgcaaaaaaacacgcttttgcacgcgggcccatacatagtacaaactctacgtttcgtagactattttacataaatagtctacaccgttgtatacgctccaaatacactaccacacattgaacctttttgcagtgcaaaaaagtacgtgtcggcagtcacgtaggccggccttatcgggtcgcgtcctgtcacgtacgaatcacattatcggaccggacgagtgttgtcttatcgtgacaggacgccagcttcctgtgttgctaaccgcagccggacgcaactccttatcggaacaggacgcgcctccatatcagccgcgcgttatctcatgcgcgtgaccggacacgaggcgcccgtcccgcttatcgcgcctataaatacagcccgcaacgatctggtaaacacagttgaacagcatctgttacagcgacacaacGGTACCgccACCatgtctagactggacaagagcaaagtcataaactctgctctggaattactcaatggagtcggtatcgaaggcctgacgacaaggaaactcgctcaaaagctgggagttgagcagcctaccctgtactggcacgtgaagaacaagcgggccctgctcgatgccctgccaatcgagatgctggacaggcatcatacccactcctgccccctggaaggcgagtcatggcaagactttctgcggaacaacgccaagtcataccgctgtgctctcctctcacatcgcgacggggctaaagtgcatctcggcacccgcccaacagagaaacagtacgaaaccctggaaaatcagctcgcgttcctgtgtcagcaaggcttctccctggagaacgcactgtacgctctgtccgccgtgggccactttacactgggctgcgtattggaggaacaggagcatcaagtagcaaaagaggaaagagagacacctaccaccgattctatgcccccacttctgaaacaagcaattgagctgttcgaccggcagggagccgaacctgccttccttttcggcctggaactaatcatatgtggcctggagaaacagctaaagtgcgaaagcggcgggccgaccgacgcccttgacgattttgacttagacatgctcccagccgatgcccttgacgactttgaccttgatatgctgcctgctgacgctcttgacgattttgaccttgacatgctccccgggtaaGGCGCGCCtttgtattgtcatgttttaatacaatatgttatgtttaaatatgtttttaataaattttataaaataatttcaacttttattgtaacaacattgtccatttacacactcctttcaagcgcgtgcgcacccgaaaagcagggtcgccgctgacgcactgctaaaaatagcacgcgcctttcaaaagcgttcacacccgaaaCTCGAGGTAAGATACATTGATGAGTTTGGACAAACCACAACTAGAATGCAGTGAAAAAAATGCTTTATTTGTGAAATTTGTGATGCTATTGCTTTATTTGTAACCATTATAAGCTGCAATAAACAAGTTAACAACAACAATTGCATTCATTTTATGTTTCAGGTTCAGGGGGAGGTGTGGGAGGTTTTTTAAAGCAAGTAAAACCTCTACAAATGTGGTATGGCTGATTATGATCGTCGACttacaatttggactttccgcccttcttggcctttatgaggatctctctgatttttcttgcgtcgagttttccggtaagacctttcggtacttcgtccacaaacacaactcctccgcgcaactttttggcggttgttacttgactggcgacgtaatccacgatctctttttccgtcatcgtctttccgtgctccaaaacaacaacggcggcgggaagttcaccggcgtcatcgtcgggaagacctgccacgcccgcgtcgaagatgttggggtgttgtaacaatatcgactccaattcagcgggggccacctggtatcctttgtatttaattaaagacttcaagcggtcaactatgaagaagtgttcgtcttcgtcccagtaagctatgtctccagaatgtagccatccatccttgtcaatcaaggcgttggtcgcttccggattgtttacataaccggacataatcataggtcctctgacacataattcgcctctctgattaacgcccagcgttttcccggtatccagatccacaaccttcgcttcaaaaaatggaacaactttaccgaccgcgcccggtttatcatccccctcgggtgtaatcagaatagctgatgtagtctcagtgagcccatatccttgtcgtatccctggaagatggaagcgttttgcaaccgcttccccgacttctttcgaaagaggtgcgcccccagaagcaatttcgtgtaaattagataaatcgtatttgtcaatcagagtgcttttggcgaagaatgaaaatagggttggtactagcaacgcactttgaattttgtaatcctgaagggatcgtaaaaacagctcttcttcaaatctatacattaagacgactcgaaatccacatatcaaatatccgagtgtagtaaacattccaaaaccgtgatggaatggaacaacacttaaaatcgcagtatccggaatgatttgattgccaaaaataggatctctggcatgcgagaatctgacgcaggcagttctatgcggaagggccacacccttaggtaacccagtagatccagaggagttcattatcagtgcaattgttttgtcacgatcaaaggactctggtacaaaatcgtattcattaaaaccgggaggtagatgagatgtgacgaacgtgtacatcgactgaaatccctggtaatccgttttagaatccatgataataattttctggattattggtaattttttttgcacgttcaaaattttttgcaacccctttttggaaacaaacactacggtaggctgcgaaatgttcatactgttgagcaattcacgttcattataaatgtcgttcgcgggcgcaactgcaactccgataaataacgcgcccaacaccggcataaagaattgaagagagttttcactgcatacgacgattctgtgatttgtattcagcccatatcgtttcatagcttctgccaaccgaacggacatttcgaagtattccgcgtacgtgatgttcacctcgatatgtgcatctgtaaaagcaattgttccaggaaccagggcgtatctcttcatagccttatgcagttgctctccagcggttccatcctctagcggatagaatggcgccgggcctttctttatgtttttggcgtcttccatggtgGCGGCCGCaagtataagacaaaagtgttgtggaattgctccaggcgatctgacggttcactaaacgagctctgcttttataggcgccCACCGTACACGCCTAAAGCTTATACGTtctctatcactgatagggagtaaactggatatacgttctctatcactgatagggagtaaactgtagatacgttctctatcactgatagggagtaaactggtcatacgttctctatcactgatagggagtaaactccttatacgttctctatcactgatagggagtaaagtctgcatacgttctctatcactgatagggagtaaactcttcatacgttctctatcactgatagggagtaaactcgaatgaagacgaaATGCATGCGTCAATTTTACGCAGACTATCTTTCTAGGGTTAATCTAGCTGCATCAGGATCATATCGTCGGGTCTTTTTTCCGGCTCAGTCATCGCCCAAGCTGGCGCTATCTGGGCATCGGGGAGGAAGAAGCCCGTGCCTTTTCCCGCGAGGTTGAAGCGGCATGGAAAGAGTTTGCCGAGGATGACTGCTGCTGCATTGACGTTGAGCGAAAACGCACGTTTACCATGATGATTCGGGAAGGTGTGGCCATGCACGCCTTTAACGGTGAACTGTTCGTTCAGGCCACCTGGGATACCAGTTCGTCGCGGCTTTTCCGGACACAGTTCCGGATGGTCAGCCCGAAGCGCATCAGCAACCCGAACAATACCGGCGACAGCCGGAACTGCCGTGCCGGTGTGCAGATTAATGACAGCGGTGCGGCGCTGGGATATTACGTCAGCGAGGACGGGTATCCTGGCTGGATGCCGCAGAAATGGACATGGATACCCCGTGAGTTACCCGGCGGGCGCGCTTGGCGTAATCATGGTCATAGCTGTTTCCTGTGTGAAATTGTTATCCGCTCACAATTCCACACAACATACGAGCCGGAAGCATAAAGTGTAAAGCCTGGGGTGCCTAATGAGTGAGCTAACTCACATTAATTGCGTTGCGCTCACTGCCCGCTTTCCAGTCGGGAAACCTGTCGTGCCAGCTGCATTAATGAATCGGCCAACGCGCGGGGAGAGGCGGTTTGCGTATTGGGCGCTCTTCCGCTTCCTCGCTCACTGACTCGCTGCGCTCGGTCGTTCGGCTGCGGCGAGCGGTATCAGCTCACTCAAAGGCGGTAATACGGTTATCCACAGAATCAGGGGATAACGCAGGAAAGAACATGTGAGCAAAAGGCCAGCAAAAGGCCAGGAACCGTAAAAAGGCCGCGTTGCTGGCGTTTTTCCATAGGCTCCGCCCCCCTGACGAGCATCACAAAAATCGACGCTCAAGTCAGAGGTGGCGAAACCCGACAGGACTATAAAGATACCAGGCGTTTCCCCCTGGAAGCTCCCTCGTGCGCTCTCCTGTTCCGACCCTGCCGCTTACCGGATACCTGTCCGCCTTTCTCCCTTCGGGAAGCGTGGCGCTTTCTCATAGCTCACGCTGTAGGTATCTCAGTTCGGTGTAGGTCGTTCGCTCCAAGCTGGGCTGTGTGCACGAACCCCCCGTTCAGCCCGACCGCTGCGCCTTATCCGGTAACTATCGTCTTGAGTCCAACCCGGTAAGACACGACTTATCGCCACTGGCAGCAGCCACTGGTAACAGGATTAGCAGAGCGAGGTATGTAGGCGGTGCTACAGAGTTCTTGAAGTGGTGGCCTAACTACGGCTACACTAGAAGGACAGTATTTGGTATCTGCGCTCTGCTGAAGCCAGTTACCTTCGGAAAAAGAGTTGGTAGCTCTTGATCCGGCAAACAAACCACCGCTGGTAGCGGTGGTTTTTTTGTTTGCAAGCAGCAGATTACGCGCAGAAAAAAAGGATCTCAAGAAGATCCTTTGATCTTTTCTACGGGGTCTGACGCTCAGTGGAACGAAAACTCACGTTAAGGGATTTTGGTCATGAGATTATCAAAAAGGATCTTCACCTAGATCCTTTTAAATTAAAAATGAAGTTTTAAATCAATCTAAAGTATATATGAGTAAACTTGGTCTGACAGTTACCAATGCTTAATCAGTGAGGCACCTATCTCAGCGATCTGTCTATTTCGTTCATCCATAGTTGCCTGACTCCCCGTCGTGTAGATAACTACGATACGGGAGGGCTTACCATCTGGCCCCAGTGCTGCAATGATACCGCGAGACCCACGCTCACCGGCTCCAGATTTATCAGCAATAAACCAGCCAGCCGGAAGGGCCGAGCGCAGAAGTGGTCCTGCAACTTTATCCGCCTCCATCCAGTCTATTAATTGTTGCCGGGAAGCTAGAGTAAGTAGTTCGCCAGTTAATAGTTTGCGCAACGTTGTTGCCATTGCTACAGGCATCGTGGTGTCACGCTCGTCGTTTGGTATGGCTTCATTCAGCTCCGGTTCCCAACGATCAAGGCGAGTTACATGATCCCCCATGTTGTGCAAAAAAGCGGTTAGCTCCTTCGGTCCTCCGATCGTTGTCAGAAGTAAGTTGGCCGCAGTGTTATCACTCATGGTTATGGCAGCACTGCATAATTCTCTTACTGTCATGCCATCCGTAAGATGCTTTTCTGTGACTGGTGAGTACTCAACCAAGTCATTCTGAGAATAGTGTATGCGGCGACCGAGTTGCTCTTGCCCGGCGTCAATACGGGATAATACCGCGCCACATAGCAGAACTTTAAAAGTGCTCATCATTGGAAAACGTTCTTCGGGGCGAAAACTCTCAAGGATCTTACCGCTGTTGAGATCCAGTTCGATGTAACCCACTCGTGCACCCAACTGATCTTCAGCATCTTTTACTTTCACCAGCGTTTCTGGGTGAGCAAAAACAGGAAGGCAAAATGCCGCAAAAAAGGGAATAAGGGCGACACGGAAATGTTGAATACTCATACTCTTCCTTTTTCAATATTATTGAAGCATTTATCAGGGTTATTGTCTCATGAGCGGATACATATTTGAATGTATTTAGAAAAATAAACAAATAGGGGTTCCGCGCACATTTCCCCGAAAAGTGCCAC

plasmid 7

CTAAATTGTAAGCGTTAATATTTTGTTAAAATTCGCGTTAAATTTTTGTTAAATCAGCTCATTTTTTAACCAATAGGCCGAAATCGGCAAAATCCCTTATAAATCAAAAGAATAGACCGAGATAGGGTTGAGTGTTGTTCCAGTTTGGAACAAGAGTCCACTATTAAAGAACGTGGACTCCAACGTCAAAGGGCGAAAAACCGTCTATCAGGGCGATGGCCCACTACGTGAACCATCACCCTAATCAAGTTTTTTGGGGTCGAGGTGCCGTAAAGCACTAAATCGGAACCCTAAAGGGAGCCCCCGATTTAGAGCTTGACGGGGAAAGCCGGCGAACGTGGCGAGAAAGGAAGGGAAGAAAGCGAAAGGAGCGGGCGCTAGGGCGCTGGCAAGTGTAGCGGTCACGCTGCGCGTAACCACCACACCCGCCGCGCTTAATGCGCCGCTACAGGGCGCGTCCCATTCGCCATTCAGGCTGCGCAACTGTTGGGAAGGGCGATCGGTGCGGGCCTCTTCGCTATTACGCCAGCTGGCGAAAGGGGGATGTGCTGCAAGGCGATTAAGTTGGGTAACGCCAGGGTTTTCCCAGTCACGACGTTGTAAAACGACGGCCAGTGAGCGCGCCTCGTTCATTCACGTTTTTGAACCCGTGGAGGACGGGCAGACTCGCGGTGCAAATGTGTTTTACAGCGTGATGGAGCAGATGAAGATGCTCGACACGCTGCAGAACACGCAGCTAGATTAACCCTAGAAAGATAATCATATTGTGACGTACGTTAAAGATAATCATGCGTAAAATTGACGCATGTGTTTTATCGGTCTGTATATCGAGGTTTATTTATTAATTTGAATAGATATTAAGTTTTATTATATTTACACTTACATACTAATAATAAATTCAACAAACAATTTATTTATGTTTATTTATTTATTAAAAAAAAACAAAAACTCAAAATTTCTTCTATAAAGTAACAAAACTTTTATCGAATTCCGCGTTACATAACTTACGGTAAATGGCCCGCCTGGCTGACCGCCCAACGACCCCCGCCCATTGACGTCAATAATGACGTATGTTCCCATAGTAACGCCAATAGGGACTTTCCATTGACGTCAATGGGTGGAGTATTTACGGTAAACTGCCCACTTGGCAGTACATCAAGTGTATCATATGCCAAGTACGCCCCCTATTGACGTCAATGACGGTAAATGGCCCGCCTGGCATTATGCCCAGTACATGACCTTATGGGACTTTCCTACTTGGCAGTACATCTACGTATTAGTCATCGCTATTACCATGGTGATGCGGTTTTGGCAGTACATCAATGGGCGTGGATAGCGGTTTGACTCACGGGGATTTCCAAGTCTCCACCCCATTGACGTCAATGGGAGTTTGTTTTGGCACCAAAATCAACGGGACTTTCCAAAATGTCGTAACAACTCCGCCCCATTGACGCAAATGGGCGGTAGGCGTGTACGGTGGGAGGTCTATATAAGCAGAGCTGGTTTAGTGAACCGTCAGATCCGCTAGCGCcaccATGGATCATTATCTGGATATTCGGCTGAGGCCTGATCCAGAGTTCCCACCTGCGCAGCTGATGTCTGTCCTTTTTGGCAAACTTCATCAGGCCCTGGTTGCCCAGGGCGGAGATCGGATAGGGGTAAGCTTTCCAGACCTCGACGAAAGCCGGAGCCGCCTGGGAGAACGCCTGCGGATCCACGCTTCTGCCGACGATCTGAGAGCCTTGCTGGCAAGGCCATGGCTTGAGGGGCTCCGGGATCACCTGCAGTTTGGCGAACCCGCCGTTGTTCCCCACCCAACCCCTTATCGGCAGGTGTCTAGAGTGCAGGCCAAATCTAATCCAGAACGGCTGCGACGGCGACTCATGCGGCGACATGATCTTAGCGAGGAAGAGGCCCGAAAAAGAATCCCTGATACCGTGGCCCGCGCCCTTGACTTGCCTTTTGTCACACTGCGGTCCCAGAGTACGGGGCAGCATTTCAGACTTTTCATTCGACACGGGCCACTGCAAGTTACCGCCGAAGAAGGAGGCTTTACTTGTTATGGACTCTCCAAGGGAGGTTTCGTGCCCTGGTTTtaaGGTACCGGGAGATGGGGGAGGCTAACTGAAACACGGAAGGAGACAATACCGGAAGGAACCCGCGCTATGACGGCAATAAAAAGACAGAATAAAACGCACGGGTGTTGGGTCGTTTGTTCactagtatcaagtgcggaaatttattaaaacacaatttatttcacaatacaattttattaagtaaagttataatgcaagtgtatatagggctacaatatagtatacatggattacaatgtgggtatacaaaagctatagtatggatataaaattattaagcacattggggttacaatataaacataatcaaccaCTTAAGtcacttgtacagctcgtccatgccgccggtggagtggcggccctcggcgcgttcgtactgttccacgatggtgtagtcctcgttgtgggaggtgatgtccaacttgatgttgacgttgtaggcgccgggcagctgcacgggcttcttggccttgtaggtggtcttgacctcagcgtcgtagtggccgccgtccttcagcttcagcctctgcttgatctcgcccttcagggcgccgtcctcggggtacatccgctcggaggaggcctcccagcccatggtTttcttctgcattacggggccgtcggaggggaagttggtgccgcgcagcttcaccttgtagatgaactcgccgtcctgcagggaggagtcctgggtcacggtcaccacgccgccgtcctcgaagttcatcacgcgctcccacttgaagccctcggggaaggacagcttcaagtagtcggggatgtcggcggggtgcttcacgtaggccttggagccgtacatgaactgaggggacaggatgtcccaggcgaagggcagggggccacccttggtcaccttcagcttggcggtctgggtgccctcgtaggggcggccctcgccctcgccctcgatctcgaactcgtggccgttcacggagccctccatgtgcaccttgaagcgcatgaactccttgatgatggccatgttatcctcctcgcccttgctcaccatggtggcaccggtcttgagtggagagagagagctagttataagcgtgtgtgtgcgctcttgtgactgaatcgaggagctaacacgggctcttatatacacaaatatgctccgcccacaacagcgattacaccatttctaggaataaatgagtaacaaggGGATCCcatgatgataaacaatgtatggtgctaatgttgcttcaacaacaattctgttgaactgtgttttcatgtttgccaacaagcacctttatactcggtggcctccccaccaccaacttttttgcactgcaaaaaaacacgcttttgcacgcgggcccatacatagtacaaactctacgtttcgtagactattttacataaatagtctacaccgttgtatacgctccaaatacactaccacacattgaacctttttgcagtgcaaaaaagtacgtgtcggcagtcacgtaggccggccttatcgggtcgcgtcctgtcacgtacgaatcacattatcggaccggacgagtgttgtcttatcgtgacaggacgccagcttcctgtgttgctaaccgcagccggacgcaactccttatcggaacaggacgcgcctccatatcagccgcgcgttatctcatgcgcgtgaccggacacgaggcgcccgtcccgcttatcgcgcctataaatacagcccgcaacgatctggtaaacacagttgaacagcatctgttacagcgacacaacGGTACCgccACCatgtctagactggacaagagcaaagtcataaactctgctctggaattactcaatggagtcggtatcgaaggcctgacgacaaggaaactcgctcaaaagctgggagttgagcagcctaccctgtactggcacgtgaagaacaagcgggccctgctcgatgccctgccaatcgagatgctggacaggcatcatacccactcctgccccctggaaggcgagtcatggcaagactttctgcggaacaacgccaagtcataccgctgtgctctcctctcacatcgcgacggggctaaagtgcatctcggcacccgcccaacagagaaacagtacgaaaccctggaaaatcagctcgcgttcctgtgtcagcaaggcttctccctggagaacgcactgtacgctctgtccgccgtgggccactttacactgggctgcgtattggaggaacaggagcatcaagtagcaaaagaggaaagagagacacctaccaccgattctatgcccccacttctgaaacaagcaattgagctgttcgaccggcagggagccgaacctgccttccttttcggcctggaactaatcatatgtggcctggagaaacagctaaagtgcgaaagcggcgggccgaccgacgcccttgacgattttgacttagacatgctcccagccgatgcccttgacgactttgaccttgatatgctgcctgctgacgctcttgacgattttgaccttgacatgctccccgggtaaGGCGCGCCtttgtattgtcatgttttaatacaatatgttatgtttaaatatgtttttaataaattttataaaataatttcaacttttattgtaacaacattgtccatttacacactcctttcaagcgcgtgcgcacccgaaaagcagggtcgccgctgacgcactgctaaaaatagcacgcgcctttcaaaagcgttcacacccgaaaCTCGAGGTAAGATACATTGATGAGTTTGGACAAACCACAACTAGAATGCAGTGAAAAAAATGCTTTATTTGTGAAATTTGTGATGCTATTGCTTTATTTGTAACCATTATAAGCTGCAATAAACAAGTTAACAACAACAATTGCATTCATTTTATGTTTCAGGTTCAGGGGGAGGTGTGGGAGGTTTTTTAAAGCAAGTAAAACCTCTACAAATGTGGTATGGCTGATTATGATCGTCGACttacaatttggactttccgcccttcttggcctttatgaggatctctctgatttttcttgcgtcgagttttccggtaagacctttcggtacttcgtccacaaacacaactcctccgcgcaactttttggcggttgttacttgactggcgacgtaatccacgatctctttttccgtcatcgtctttccgtgctccaaaacaacaacggcggcgggaagttcaccggcgtcatcgtcgggaagacctgccacgcccgcgtcgaagatgttggggtgttgtaacaatatcgactccaattcagcgggggccacctggtatcctttgtatttaattaaagacttcaagcggtcaactatgaagaagtgttcgtcttcgtcccagtaagctatgtctccagaatgtagccatccatccttgtcaatcaaggcgttggtcgcttccggattgtttacataaccggacataatcataggtcctctgacacataattcgcctctctgattaacgcccagcgttttcccggtatccagatccacaaccttcgcttcaaaaaatggaacaactttaccgaccgcgcccggtttatcatccccctcgggtgtaatcagaatagctgatgtagtctcagtgagcccatatccttgtcgtatccctggaagatggaagcgttttgcaaccgcttccccgacttctttcgaaagaggtgcgcccccagaagcaatttcgtgtaaattagataaatcgtatttgtcaatcagagtgcttttggcgaagaatgaaaatagggttggtactagcaacgcactttgaattttgtaatcctgaagggatcgtaaaaacagctcttcttcaaatctatacattaagacgactcgaaatccacatatcaaatatccgagtgtagtaaacattccaaaaccgtgatggaatggaacaacacttaaaatcgcagtatccggaatgatttgattgccaaaaataggatctctggcatgcgagaatctgacgcaggcagttctatgcggaagggccacacccttaggtaacccagtagatccagaggagttcattatcagtgcaattgttttgtcacgatcaaaggactctggtacaaaatcgtattcattaaaaccgggaggtagatgagatgtgacgaacgtgtacatcgactgaaatccctggtaatccgttttagaatccatgataataattttctggattattggtaattttttttgcacgttcaaaattttttgcaacccctttttggaaacaaacactacggtaggctgcgaaatgttcatactgttgagcaattcacgttcattataaatgtcgttcgcgggcgcaactgcaactccgataaataacgcgcccaacaccggcataaagaattgaagagagttttcactgcatacgacgattctgtgatttgtattcagcccatatcgtttcatagcttctgccaaccgaacggacatttcgaagtattccgcgtacgtgatgttcacctcgatatgtgcatctgtaaaagcaattgttccaggaaccagggcgtatctcttcatagccttatgcagttgctctccagcggttccatcctctagcggatagaatggcgccgggcctttctttatgtttttggcgtcttccatggtgGCGGCCGCTTTCTTAGCTGCCTATACGGCAGTGAACaagtataagacaaaagtgttgtggaattgctccaggcgatctgacggttcactaaacgagctctgcttttataggcgccCACCGTACACGCCTAAAGCTTATACGTtctctatcactgatagggagtaaactggatatacgttctctatcactgatagggagtaaactgtagatacgttctctatcactgatagggagtaaactggtcatacgttctctatcactgatagggagtaaactccttatacgttctctatcactgatagggagtaaagtctgcatacgttctctatcactgatagggagtaaactcttcatacgttctctatcactgatagggagtaaactcgaatgaagacgaaATGCATGCGTCAATTTTACGCAGACTATCTTTCTAGGGTTAATCTAGCTGCATCAGGATCATATCGTCGGGTCTTTTTTCCGGCTCAGTCATCGCCCAAGCTGGCGCTATCTGGGCATCGGGGAGGAAGAAGCCCGTGCCTTTTCCCGCGAGGTTGAAGCGGCATGGAAAGAGTTTGCCGAGGATGACTGCTGCTGCATTGACGTTGAGCGAAAACGCACGTTTACCATGATGATTCGGGAAGGTGTGGCCATGCACGCCTTTAACGGTGAACTGTTCGTTCAGGCCACCTGGGATACCAGTTCGTCGCGGCTTTTCCGGACACAGTTCCGGATGGTCAGCCCGAAGCGCATCAGCAACCCGAACAATACCGGCGACAGCCGGAACTGCCGTGCCGGTGTGCAGATTAATGACAGCGGTGCGGCGCTGGGATATTACGTCAGCGAGGACGGGTATCCTGGCTGGATGCCGCAGAAATGGACATGGATACCCCGTGAGTTACCCGGCGGGCGCGCTTGGCGTAATCATGGTCATAGCTGTTTCCTGTGTGAAATTGTTATCCGCTCACAATTCCACACAACATACGAGCCGGAAGCATAAAGTGTAAAGCCTGGGGTGCCTAATGAGTGAGCTAACTCACATTAATTGCGTTGCGCTCACTGCCCGCTTTCCAGTCGGGAAACCTGTCGTGCCAGCTGCATTAATGAATCGGCCAACGCGCGGGGAGAGGCGGTTTGCGTATTGGGCGCTCTTCCGCTTCCTCGCTCACTGACTCGCTGCGCTCGGTCGTTCGGCTGCGGCGAGCGGTATCAGCTCACTCAAAGGCGGTAATACGGTTATCCACAGAATCAGGGGATAACGCAGGAAAGAACATGTGAGCAAAAGGCCAGCAAAAGGCCAGGAACCGTAAAAAGGCCGCGTTGCTGGCGTTTTTCCATAGGCTCCGCCCCCCTGACGAGCATCACAAAAATCGACGCTCAAGTCAGAGGTGGCGAAACCCGACAGGACTATAAAGATACCAGGCGTTTCCCCCTGGAAGCTCCCTCGTGCGCTCTCCTGTTCCGACCCTGCCGCTTACCGGATACCTGTCCGCCTTTCTCCCTTCGGGAAGCGTGGCGCTTTCTCATAGCTCACGCTGTAGGTATCTCAGTTCGGTGTAGGTCGTTCGCTCCAAGCTGGGCTGTGTGCACGAACCCCCCGTTCAGCCCGACCGCTGCGCCTTATCCGGTAACTATCGTCTTGAGTCCAACCCGGTAAGACACGACTTATCGCCACTGGCAGCAGCCACTGGTAACAGGATTAGCAGAGCGAGGTATGTAGGCGGTGCTACAGAGTTCTTGAAGTGGTGGCCTAACTACGGCTACACTAGAAGGACAGTATTTGGTATCTGCGCTCTGCTGAAGCCAGTTACCTTCGGAAAAAGAGTTGGTAGCTCTTGATCCGGCAAACAAACCACCGCTGGTAGCGGTGGTTTTTTTGTTTGCAAGCAGCAGATTACGCGCAGAAAAAAAGGATCTCAAGAAGATCCTTTGATCTTTTCTACGGGGTCTGACGCTCAGTGGAACGAAAACTCACGTTAAGGGATTTTGGTCATGAGATTATCAAAAAGGATCTTCACCTAGATCCTTTTAAATTAAAAATGAAGTTTTAAATCAATCTAAAGTATATATGAGTAAACTTGGTCTGACAGTTACCAATGCTTAATCAGTGAGGCACCTATCTCAGCGATCTGTCTATTTCGTTCATCCATAGTTGCCTGACTCCCCGTCGTGTAGATAACTACGATACGGGAGGGCTTACCATCTGGCCCCAGTGCTGCAATGATACCGCGAGACCCACGCTCACCGGCTCCAGATTTATCAGCAATAAACCAGCCAGCCGGAAGGGCCGAGCGCAGAAGTGGTCCTGCAACTTTATCCGCCTCCATCCAGTCTATTAATTGTTGCCGGGAAGCTAGAGTAAGTAGTTCGCCAGTTAATAGTTTGCGCAACGTTGTTGCCATTGCTACAGGCATCGTGGTGTCACGCTCGTCGTTTGGTATGGCTTCATTCAGCTCCGGTTCCCAACGATCAAGGCGAGTTACATGATCCCCCATGTTGTGCAAAAAAGCGGTTAGCTCCTTCGGTCCTCCGATCGTTGTCAGAAGTAAGTTGGCCGCAGTGTTATCACTCATGGTTATGGCAGCACTGCATAATTCTCTTACTGTCATGCCATCCGTAAGATGCTTTTCTGTGACTGGTGAGTACTCAACCAAGTCATTCTGAGAATAGTGTATGCGGCGACCGAGTTGCTCTTGCCCGGCGTCAATACGGGATAATACCGCGCCACATAGCAGAACTTTAAAAGTGCTCATCATTGGAAAACGTTCTTCGGGGCGAAAACTCTCAAGGATCTTACCGCTGTTGAGATCCAGTTCGATGTAACCCACTCGTGCACCCAACTGATCTTCAGCATCTTTTACTTTCACCAGCGTTTCTGGGTGAGCAAAAACAGGAAGGCAAAATGCCGCAAAAAAGGGAATAAGGGCGACACGGAAATGTTGAATACTCATACTCTTCCTTTTTCAATATTATTGAAGCATTTATCAGGGTTATTGTCTCATGAGCGGATACATATTTGAATGTATTTAGAAAAATAAACAAATAGGGGTTCCGCGCACATTTCCCCGAAAAGTGCCAC

plasmid 8

CTAAATTGTAAGCGTTAATATTTTGTTAAAATTCGCGTTAAATTTTTGTTAAATCAGCTCATTTTTTAACCAATAGGCCGAAATCGGCAAAATCCCTTATAAATCAAAAGAATAGACCGAGATAGGGTTGAGTGTTGTTCCAGTTTGGAACAAGAGTCCACTATTAAAGAACGTGGACTCCAACGTCAAAGGGCGAAAAACCGTCTATCAGGGCGATGGCCCACTACGTGAACCATCACCCTAATCAAGTTTTTTGGGGTCGAGGTGCCGTAAAGCACTAAATCGGAACCCTAAAGGGAGCCCCCGATTTAGAGCTTGACGGGGAAAGCCGGCGAACGTGGCGAGAAAGGAAGGGAAGAAAGCGAAAGGAGCGGGCGCTAGGGCGCTGGCAAGTGTAGCGGTCACGCTGCGCGTAACCACCACACCCGCCGCGCTTAATGCGCCGCTACAGGGCGCGTCCCATTCGCCATTCAGGCTGCGCAACTGTTGGGAAGGGCGATCGGTGCGGGCCTCTTCGCTATTACGCCAGCTGGCGAAAGGGGGATGTGCTGCAAGGCGATTAAGTTGGGTAACGCCAGGGTTTTCCCAGTCACGACGTTGTAAAACGACGGCCAGTGAGCGCGCCTCGTTCATTCACGTTTTTGAACCCGTGGAGGACGGGCAGACTCGCGGTGCAAATGTGTTTTACAGCGTGATGGAGCAGATGAAGATGCTCGACACGCTGCAGAACACGCAGCTAGATTAACCCTAGAAAGATAATCATATTGTGACGTACGTTAAAGATAATCATGCGTAAAATTGACGCATGTGTTTTATCGGTCTGTATATCGAGGTTTATTTATTAATTTGAATAGATATTAAGTTTTATTATATTTACACTTACATACTAATAATAAATTCAACAAACAATTTATTTATGTTTATTTATTTATTAAAAAAAAACAAAAACTCAAAATTTCTTCTATAAAGTAACAAAACTTTTATCGAATTCAAATGGTGTAATCGCTGTTGTGGGCGGAGCATATTTGTGTATATAAGAGCCCGTGTTAGCTCCTCGATTCAGTCACAAGAGCGCACACACACGCTTATAACTAGCTCTCTCTCTCCACTCAAGGCTAGCGCcaccATGGATCATTATCTGGATATTCGGCTGAGGCCTGATCCAGAGTTCCCACCTGCGCAGCTGATGTCTGTCCTTTTTGGCAAACTTCATCAGGCCCTGGTTGCCCAGGGCGGAGATCGGATAGGGGTAAGCTTTCCAGACCTCGACGAAAGCCGGAGCCGCCTGGGAGAACGCCTGCGGATCCACGCTTCTGCCGACGATCTGAGAGCCTTGCTGGCAAGGCCATGGCTTGAGGGGCTCCGGGATCACCTGCAGTTTGGCGAACCCGCCGTTGTTCCCCACCCAACCCCTTATCGGCAGGTGTCTAGAGTGCAGGCCAAATCTAATCCAGAACGGCTGCGACGGCGACTCATGCGGCGACATGATCTTAGCGAGGAAGAGGCCCGAAAAAGAATCCCTGATACCGTGGCCCGCGCCCTTGACTTGCCTTTTGTCACACTGCGGTCCCAGAGTACGGGGCAGCATTTCAGACTTTTCATTCGACACGGGCCACTGCAAGTTACCGCCGAAGAAGGAGGCTTTACTTGTTATGGACTCTCCAAGGGAGGTTTCGTGCCCTGGTTTtaaGGTACCGGGAGATGGGGGAGGCTAACTGAAACACGGAAGGAGACAATACCGGAAGGAACCCGCGCTATGACGGCAATAAAAAGACAGAATAAAACGCACGGGTGTTGGGTCGTTTGTTCactagtatcaagtgcggaaatttattaaaacacaatttatttcacaatacaattttattaagtaaagttataatgcaagtgtatatagggctacaatatagtatacatggattacaatgtgggtatacaaaagctatagtatggatataaaattattaagcacattggggttacaatataaacataatcaaccaCTTAAGtcacttgtacagctcgtccatgccgccggtggagtggcggccctcggcgcgttcgtactgttccacgatggtgtagtcctcgttgtgggaggtgatgtccaacttgatgttgacgttgtaggcgccgggcagctgcacgggcttcttggccttgtaggtggtcttgacctcagcgtcgtagtggccgccgtccttcagcttcagcctctgcttgatctcgcccttcagggcgccgtcctcggggtacatccgctcggaggaggcctcccagcccatggtTttcttctgcattacggggccgtcggaggggaagttggtgccgcgcagcttcaccttgtagatgaactcgccgtcctgcagggaggagtcctgggtcacggtcaccacgccgccgtcctcgaagttcatcacgcgctcccacttgaagccctcggggaaggacagcttcaagtagtcggggatgtcggcggggtgcttcacgtaggccttggagccgtacatgaactgaggggacaggatgtcccaggcgaagggcagggggccacccttggtcaccttcagcttggcggtctgggtgccctcgtaggggcggccctcgccctcgccctcgatctcgaactcgtggccgttcacggagccctccatgtgcaccttgaagcgcatgaactccttgatgatggccatgttatcctcctcgcccttgctcaccatggtggcaccggtcttgagtggagagagagagctagttataagcgtgtgtgtgcgctcttgtgactgaatcgaggagctaacacgggctcttatatacacaaatatgctccgcccacaacagcgattacaccatttctaggaataaatgagtaacaaggGGATCCcatgatgataaacaatgtatggtgctaatgttgcttcaacaacaattctgttgaactgtgttttcatgtttgccaacaagcacctttatactcggtggcctccccaccaccaacttttttgcactgcaaaaaaacacgcttttgcacgcgggcccatacatagtacaaactctacgtttcgtagactattttacataaatagtctacaccgttgtatacgctccaaatacactaccacacattgaacctttttgcagtgcaaaaaagtacgtgtcggcagtcacgtaggccggccttatcgggtcgcgtcctgtcacgtacgaatcacattatcggaccggacgagtgttgtcttatcgtgacaggacgccagcttcctgtgttgctaaccgcagccggacgcaactccttatcggaacaggacgcgcctccatatcagccgcgcgttatctcatgcgcgtgaccggacacgaggcgcccgtcccgcttatcgcgcctataaatacagcccgcaacgatctggtaaacacagttgaacagcatctgttacagcgacacaacGGTACCgccACCatgtctagactggacaagagcaaagtcataaactctgctctggaattactcaatggagtcggtatcgaaggcctgacgacaaggaaactcgctcaaaagctgggagttgagcagcctaccctgtactggcacgtgaagaacaagcgggccctgctcgatgccctgccaatcgagatgctggacaggcatcatacccactcctgccccctggaaggcgagtcatggcaagactttctgcggaacaacgccaagtcataccgctgtgctctcctctcacatcgcgacggggctaaagtgcatctcggcacccgcccaacagagaaacagtacgaaaccctggaaaatcagctcgcgttcctgtgtcagcaaggcttctccctggagaacgcactgtacgctctgtccgccgtgggccactttacactgggctgcgtattggaggaacaggagcatcaagtagcaaaagaggaaagagagacacctaccaccgattctatgcccccacttctgaaacaagcaattgagctgttcgaccggcagggagccgaacctgccttccttttcggcctggaactaatcatatgtggcctggagaaacagctaaagtgcgaaagcggcgggccgaccgacgcccttgacgattttgacttagacatgctcccagccgatgcccttgacgactttgaccttgatatgctgcctgctgacgctcttgacgattttgaccttgacatgctccccgggtaaGGCGCGCCtttgtattgtcatgttttaatacaatatgttatgtttaaatatgtttttaataaattttataaaataatttcaacttttattgtaacaacattgtccatttacacactcctttcaagcgcgtgcgcacccgaaaagcagggtcgccgctgacgcactgctaaaaatagcacgcgcctttcaaaagcgttcacacccgaaaCTCGAGGTAAGATACATTGATGAGTTTGGACAAACCACAACTAGAATGCAGTGAAAAAAATGCTTTATTTGTGAAATTTGTGATGCTATTGCTTTATTTGTAACCATTATAAGCTGCAATAAACAAGTTAACAACAACAATTGCATTCATTTTATGTTTCAGGTTCAGGGGGAGGTGTGGGAGGTTTTTTAAAGCAAGTAAAACCTCTACAAATGTGGTATGGCTGATTATGATCGTCGACttacaatttggactttccgcccttcttggcctttatgaggatctctctgatttttcttgcgtcgagttttccggtaagacctttcggtacttcgtccacaaacacaactcctccgcgcaactttttggcggttgttacttgactggcgacgtaatccacgatctctttttccgtcatcgtctttccgtgctccaaaacaacaacggcggcgggaagttcaccggcgtcatcgtcgggaagacctgccacgcccgcgtcgaagatgttggggtgttgtaacaatatcgactccaattcagcgggggccacctggtatcctttgtatttaattaaagacttcaagcggtcaactatgaagaagtgttcgtcttcgtcccagtaagctatgtctccagaatgtagccatccatccttgtcaatcaaggcgttggtcgcttccggattgtttacataaccggacataatcataggtcctctgacacataattcgcctctctgattaacgcccagcgttttcccggtatccagatccacaaccttcgcttcaaaaaatggaacaactttaccgaccgcgcccggtttatcatccccctcgggtgtaatcagaatagctgatgtagtctcagtgagcccatatccttgtcgtatccctggaagatggaagcgttttgcaaccgcttccccgacttctttcgaaagaggtgcgcccccagaagcaatttcgtgtaaattagataaatcgtatttgtcaatcagagtgcttttggcgaagaatgaaaatagggttggtactagcaacgcactttgaattttgtaatcctgaagggatcgtaaaaacagctcttcttcaaatctatacattaagacgactcgaaatccacatatcaaatatccgagtgtagtaaacattccaaaaccgtgatggaatggaacaacacttaaaatcgcagtatccggaatgatttgattgccaaaaataggatctctggcatgcgagaatctgacgcaggcagttctatgcggaagggccacacccttaggtaacccagtagatccagaggagttcattatcagtgcaattgttttgtcacgatcaaaggactctggtacaaaatcgtattcattaaaaccgggaggtagatgagatgtgacgaacgtgtacatcgactgaaatccctggtaatccgttttagaatccatgataataattttctggattattggtaattttttttgcacgttcaaaattttttgcaacccctttttggaaacaaacactacggtaggctgcgaaatgttcatactgttgagcaattcacgttcattataaatgtcgttcgcgggcgcaactgcaactccgataaataacgcgcccaacaccggcataaagaattgaagagagttttcactgcatacgacgattctgtgatttgtattcagcccatatcgtttcatagcttctgccaaccgaacggacatttcgaagtattccgcgtacgtgatgttcacctcgatatgtgcatctgtaaaagcaattgttccaggaaccagggcgtatctcttcatagccttatgcagttgctctccagcggttccatcctctagcggatagaatggcgccgggcctttctttatgtttttggcgtcttccatggtgGCGGCCGCTTTCTTAGCTGCCTATACGGCAGTGAACaagtataagacaaaagtgttgtggaattgctccaggcgatctgacggttcactaaacgagctctgcttttataggcgccCACCGTACACGCCTAAAGCTTATACGTtctctatcactgatagggagtaaactggatatacgttctctatcactgatagggagtaaactgtagatacgttctctatcactgatagggagtaaactggtcatacgttctctatcactgatagggagtaaactccttatacgttctctatcactgatagggagtaaagtctgcatacgttctctatcactgatagggagtaaactcttcatacgttctctatcactgatagggagtaaactcgaatgaagacgaaATGCATGCGTCAATTTTACGCAGACTATCTTTCTAGGGTTAATCTAGCTGCATCAGGATCATATCGTCGGGTCTTTTTTCCGGCTCAGTCATCGCCCAAGCTGGCGCTATCTGGGCATCGGGGAGGAAGAAGCCCGTGCCTTTTCCCGCGAGGTTGAAGCGGCATGGAAAGAGTTTGCCGAGGATGACTGCTGCTGCATTGACGTTGAGCGAAAACGCACGTTTACCATGATGATTCGGGAAGGTGTGGCCATGCACGCCTTTAACGGTGAACTGTTCGTTCAGGCCACCTGGGATACCAGTTCGTCGCGGCTTTTCCGGACACAGTTCCGGATGGTCAGCCCGAAGCGCATCAGCAACCCGAACAATACCGGCGACAGCCGGAACTGCCGTGCCGGTGTGCAGATTAATGACAGCGGTGCGGCGCTGGGATATTACGTCAGCGAGGACGGGTATCCTGGCTGGATGCCGCAGAAATGGACATGGATACCCCGTGAGTTACCCGGCGGGCGCGCTTGGCGTAATCATGGTCATAGCTGTTTCCTGTGTGAAATTGTTATCCGCTCACAATTCCACACAACATACGAGCCGGAAGCATAAAGTGTAAAGCCTGGGGTGCCTAATGAGTGAGCTAACTCACATTAATTGCGTTGCGCTCACTGCCCGCTTTCCAGTCGGGAAACCTGTCGTGCCAGCTGCATTAATGAATCGGCCAACGCGCGGGGAGAGGCGGTTTGCGTATTGGGCGCTCTTCCGCTTCCTCGCTCACTGACTCGCTGCGCTCGGTCGTTCGGCTGCGGCGAGCGGTATCAGCTCACTCAAAGGCGGTAATACGGTTATCCACAGAATCAGGGGATAACGCAGGAAAGAACATGTGAGCAAAAGGCCAGCAAAAGGCCAGGAACCGTAAAAAGGCCGCGTTGCTGGCGTTTTTCCATAGGCTCCGCCCCCCTGACGAGCATCACAAAAATCGACGCTCAAGTCAGAGGTGGCGAAACCCGACAGGACTATAAAGATACCAGGCGTTTCCCCCTGGAAGCTCCCTCGTGCGCTCTCCTGTTCCGACCCTGCCGCTTACCGGATACCTGTCCGCCTTTCTCCCTTCGGGAAGCGTGGCGCTTTCTCATAGCTCACGCTGTAGGTATCTCAGTTCGGTGTAGGTCGTTCGCTCCAAGCTGGGCTGTGTGCACGAACCCCCCGTTCAGCCCGACCGCTGCGCCTTATCCGGTAACTATCGTCTTGAGTCCAACCCGGTAAGACACGACTTATCGCCACTGGCAGCAGCCACTGGTAACAGGATTAGCAGAGCGAGGTATGTAGGCGGTGCTACAGAGTTCTTGAAGTGGTGGCCTAACTACGGCTACACTAGAAGGACAGTATTTGGTATCTGCGCTCTGCTGAAGCCAGTTACCTTCGGAAAAAGAGTTGGTAGCTCTTGATCCGGCAAACAAACCACCGCTGGTAGCGGTGGTTTTTTTGTTTGCAAGCAGCAGATTACGCGCAGAAAAAAAGGATCTCAAGAAGATCCTTTGATCTTTTCTACGGGGTCTGACGCTCAGTGGAACGAAAACTCACGTTAAGGGATTTTGGTCATGAGATTATCAAAAAGGATCTTCACCTAGATCCTTTTAAATTAAAAATGAAGTTTTAAATCAATCTAAAGTATATATGAGTAAACTTGGTCTGACAGTTACCAATGCTTAATCAGTGAGGCACCTATCTCAGCGATCTGTCTATTTCGTTCATCCATAGTTGCCTGACTCCCCGTCGTGTAGATAACTACGATACGGGAGGGCTTACCATCTGGCCCCAGTGCTGCAATGATACCGCGAGACCCACGCTCACCGGCTCCAGATTTATCAGCAATAAACCAGCCAGCCGGAAGGGCCGAGCGCAGAAGTGGTCCTGCAACTTTATCCGCCTCCATCCAGTCTATTAATTGTTGCCGGGAAGCTAGAGTAAGTAGTTCGCCAGTTAATAGTTTGCGCAACGTTGTTGCCATTGCTACAGGCATCGTGGTGTCACGCTCGTCGTTTGGTATGGCTTCATTCAGCTCCGGTTCCCAACGATCAAGGCGAGTTACATGATCCCCCATGTTGTGCAAAAAAGCGGTTAGCTCCTTCGGTCCTCCGATCGTTGTCAGAAGTAAGTTGGCCGCAGTGTTATCACTCATGGTTATGGCAGCACTGCATAATTCTCTTACTGTCATGCCATCCGTAAGATGCTTTTCTGTGACTGGTGAGTACTCAACCAAGTCATTCTGAGAATAGTGTATGCGGCGACCGAGTTGCTCTTGCCCGGCGTCAATACGGGATAATACCGCGCCACATAGCAGAACTTTAAAAGTGCTCATCATTGGAAAACGTTCTTCGGGGCGAAAACTCTCAAGGATCTTACCGCTGTTGAGATCCAGTTCGATGTAACCCACTCGTGCACCCAACTGATCTTCAGCATCTTTTACTTTCACCAGCGTTTCTGGGTGAGCAAAAACAGGAAGGCAAAATGCCGCAAAAAAGGGAATAAGGGCGACACGGAAATGTTGAATACTCATACTCTTCCTTTTTCAATATTATTGAAGCATTTATCAGGGTTATTGTCTCATGAGCGGATACATATTTGAATGTATTTAGAAAAATAAACAAATAGGGGTTCCGCGCACATTTCCCCGAAAAGTGCCAC

plasmid 9

CTAAATTGTAAGCGTTAATATTTTGTTAAAATTCGCGTTAAATTTTTGTTAAATCAGCTCATTTTTTAACCAATAGGCCGAAATCGGCAAAATCCCTTATAAATCAAAAGAATAGACCGAGATAGGGTTGAGTGTTGTTCCAGTTTGGAACAAGAGTCCACTATTAAAGAACGTGGACTCCAACGTCAAAGGGCGAAAAACCGTCTATCAGGGCGATGGCCCACTACGTGAACCATCACCCTAATCAAGTTTTTTGGGGTCGAGGTGCCGTAAAGCACTAAATCGGAACCCTAAAGGGAGCCCCCGATTTAGAGCTTGACGGGGAAAGCCGGCGAACGTGGCGAGAAAGGAAGGGAAGAAAGCGAAAGGAGCGGGCGCTAGGGCGCTGGCAAGTGTAGCGGTCACGCTGCGCGTAACCACCACACCCGCCGCGCTTAATGCGCCGCTACAGGGCGCGTCCCATTCGCCATTCAGGCTGCGCAACTGTTGGGAAGGGCGATCGGTGCGGGCCTCTTCGCTATTACGCCAGCTGGCGAAAGGGGGATGTGCTGCAAGGCGATTAAGTTGGGTAACGCCAGGGTTTTCCCAGTCACGACGTTGTAAAACGACGGCCAGTGAGCGCGCCTCGTTCATTCACGTTTTTGAACCCGTGGAGGACGGGCAGACTCGCGGTGCAAATGTGTTTTACAGCGTGATGGAGCAGATGAAGATGCTCGACACGCTGCAGAACACGCAGCTAGATTAACCCTAGAAAGATAATCATATTGTGACGTACGTTAAAGATAATCATGCGTAAAATTGACGCATGTGTTTTATCGGTCTGTATATCGAGGTTTATTTATTAATTTGAATAGATATTAAGTTTTATTATATTTACACTTACATACTAATAATAAATTCAACAAACAATTTATTTATGTTTATTTATTTATTAAAAAAAAACAAAAACTCAAAATTTCTTCTATAAAGTAACAAAACTTTTATCGAATTCGATGTCTTTGTGATGCGCGCGACATTTTTGTAGGTTATTGATAAAATGAACGGATACGTTGCCCGACATTATCATTAAATCCTTGGCGTAGAATTTGTCGGGTCCATTGTCCGTGTGCGCTAGCATGCCCGTAACGGACCTCGTACTTTTGGCTTCAAAGGTTTTGCGCACAGACAAAATGTGCCACACTTGCAGCTCTGCATGTGTGCGCGTTACCACAAATCCCAACGGCGCAGTGTACTTGTTGTATGCAAATAAATCTCGATAAAGGCGCGGCGCGCGAATGCAGCTGATCACGTACGCTCCTCGTGTTCCGTTCAAGGACGGTGTTATCGACCTCAGATTAATGTTTATCGGCCGACTGTTTTCGTATCCGCTCACCAAACGCGTTTTTGCATTAACATTGTATGTCGGCGGATGTTCTATATCTAATTTGAATAAATAAACGATAACCGCGTTGGTTTTAGAGGGCATAATAAAAGAAATATTGTTATCGTGTTCGCCATTAGGGCAGTATAAATTGACGTTCATGTTGGATATTGTTTCAGTTGCAAGTTGACACTGGCGGCGACAAGATCGTGAACAACCAAGTGACTATTACGCAAATTAATTTTAACGCGGCcaccATGGATCATTATCTGGATATTCGGCTGAGGCCTGATCCAGAGTTCCCACCTGCGCAGCTGATGTCTGTCCTTTTTGGCAAACTTCATCAGGCCCTGGTTGCCCAGGGCGGAGATCGGATAGGGGTAAGCTTTCCAGACCTCGACGAAAGCCGGAGCCGCCTGGGAGAACGCCTGCGGATCCACGCTTCTGCCGACGATCTGAGAGCCTTGCTGGCAAGGCCATGGCTTGAGGGGCTCCGGGATCACCTGCAGTTTGGCGAACCCGCCGTTGTTCCCCACCCAACCCCTTATCGGCAGGTGTCTAGAGTGCAGGCCAAATCTAATCCAGAACGGCTGCGACGGCGACTCATGCGGCGACATGATCTTAGCGAGGAAGAGGCCCGAAAAAGAATCCCTGATACCGTGGCCCGCGCCCTTGACTTGCCTTTTGTCACACTGCGGTCCCAGAGTACGGGGCAGCATTTCAGACTTTTCATTCGACACGGGCCACTGCAAGTTACCGCCGAAGAAGGAGGCTTTACTTGTTATGGACTCTCCAAGGGAGGTTTCGTGCCCTGGTTTtaaGGTACCGGGAGATGGGGGAGGCTAACTGAAACACGGAAGGAGACAATACCGGAAGGAACCCGCGCTATGACGGCAATAAAAAGACAGAATAAAACGCACGGGTGTTGGGTCGTTTGTTCactagtatcaagtgcggaaatttattaaaacacaatttatttcacaatacaattttattaagtaaagttataatgcaagtgtatatagggctacaatatagtatacatggattacaatgtgggtatacaaaagctatagtatggatataaaattattaagcacattggggttacaatataaacataatcaaccaCTTAAGtcacttgtacagctcgtccatgccgccggtggagtggcggccctcggcgcgttcgtactgttccacgatggtgtagtcctcgttgtgggaggtgatgtccaacttgatgttgacgttgtaggcgccgggcagctgcacgggcttcttggccttgtaggtggtcttgacctcagcgtcgtagtggccgccgtccttcagcttcagcctctgcttgatctcgcccttcagggcgccgtcctcggggtacatccgctcggaggaggcctcccagcccatggtTttcttctgcattacggggccgtcggaggggaagttggtgccgcgcagcttcaccttgtagatgaactcgccgtcctgcagggaggagtcctgggtcacggtcaccacgccgccgtcctcgaagttcatcacgcgctcccacttgaagccctcggggaaggacagcttcaagtagtcggggatgtcggcggggtgcttcacgtaggccttggagccgtacatgaactgaggggacaggatgtcccaggcgaagggcagggggccacccttggtcaccttcagcttggcggtctgggtgccctcgtaggggcggccctcgccctcgccctcgatctcgaactcgtggccgttcacggagccctccatgtgcaccttgaagcgcatgaactccttgatgatggccatgttatcctcctcgcccttgctcaccatggtggcaccggtcttgagtggagagagagagctagttataagcgtgtgtgtgcgctcttgtgactgaatcgaggagctaacacgggctcttatatacacaaatatgctccgcccacaacagcgattacaccatttctaggaataaatgagtaacaaggGGATCCcatgatgataaacaatgtatggtgctaatgttgcttcaacaacaattctgttgaactgtgttttcatgtttgccaacaagcacctttatactcggtggcctccccaccaccaacttttttgcactgcaaaaaaacacgcttttgcacgcgggcccatacatagtacaaactctacgtttcgtagactattttacataaatagtctacaccgttgtatacgctccaaatacactaccacacattgaacctttttgcagtgcaaaaaagtacgtgtcggcagtcacgtaggccggccttatcgggtcgcgtcctgtcacgtacgaatcacattatcggaccggacgagtgttgtcttatcgtgacaggacgccagcttcctgtgttgctaaccgcagccggacgcaactccttatcggaacaggacgcgcctccatatcagccgcgcgttatctcatgcgcgtgaccggacacgaggcgcccgtcccgcttatcgcgcctataaatacagcccgcaacgatctggtaaacacagttgaacagcatctgttacagcgacacaacGGTACCgccACCatgtctagactggacaagagcaaagtcataaactctgctctggaattactcaatggagtcggtatcgaaggcctgacgacaaggaaactcgctcaaaagctgggagttgagcagcctaccctgtactggcacgtgaagaacaagcgggccctgctcgatgccctgccaatcgagatgctggacaggcatcatacccactcctgccccctggaaggcgagtcatggcaagactttctgcggaacaacgccaagtcataccgctgtgctctcctctcacatcgcgacggggctaaagtgcatctcggcacccgcccaacagagaaacagtacgaaaccctggaaaatcagctcgcgttcctgtgtcagcaaggcttctccctggagaacgcactgtacgctctgtccgccgtgggccactttacactgggctgcgtattggaggaacaggagcatcaagtagcaaaagaggaaagagagacacctaccaccgattctatgcccccacttctgaaacaagcaattgagctgttcgaccggcagggagccgaacctgccttccttttcggcctggaactaatcatatgtggcctggagaaacagctaaagtgcgaaagcggcgggccgaccgacgcccttgacgattttgacttagacatgctcccagccgatgcccttgacgactttgaccttgatatgctgcctgctgacgctcttgacgattttgaccttgacatgctccccgggtaaGGCGCGCCtttgtattgtcatgttttaatacaatatgttatgtttaaatatgtttttaataaattttataaaataatttcaacttttattgtaacaacattgtccatttacacactcctttcaagcgcgtgcgcacccgaaaagcagggtcgccgctgacgcactgctaaaaatagcacgcgcctttcaaaagcgttcacacccgaaaCTCGAGGTAAGATACATTGATGAGTTTGGACAAACCACAACTAGAATGCAGTGAAAAAAATGCTTTATTTGTGAAATTTGTGATGCTATTGCTTTATTTGTAACCATTATAAGCTGCAATAAACAAGTTAACAACAACAATTGCATTCATTTTATGTTTCAGGTTCAGGGGGAGGTGTGGGAGGTTTTTTAAAGCAAGTAAAACCTCTACAAATGTGGTATGGCTGATTATGATCGTCGACttacaatttggactttccgcccttcttggcctttatgaggatctctctgatttttcttgcgtcgagttttccggtaagacctttcggtacttcgtccacaaacacaactcctccgcgcaactttttggcggttgttacttgactggcgacgtaatccacgatctctttttccgtcatcgtctttccgtgctccaaaacaacaacggcggcgggaagttcaccggcgtcatcgtcgggaagacctgccacgcccgcgtcgaagatgttggggtgttgtaacaatatcgactccaattcagcgggggccacctggtatcctttgtatttaattaaagacttcaagcggtcaactatgaagaagtgttcgtcttcgtcccagtaagctatgtctccagaatgtagccatccatccttgtcaatcaaggcgttggtcgcttccggattgtttacataaccggacataatcataggtcctctgacacataattcgcctctctgattaacgcccagcgttttcccggtatccagatccacaaccttcgcttcaaaaaatggaacaactttaccgaccgcgcccggtttatcatccccctcgggtgtaatcagaatagctgatgtagtctcagtgagcccatatccttgtcgtatccctggaagatggaagcgttttgcaaccgcttccccgacttctttcgaaagaggtgcgcccccagaagcaatttcgtgtaaattagataaatcgtatttgtcaatcagagtgcttttggcgaagaatgaaaatagggttggtactagcaacgcactttgaattttgtaatcctgaagggatcgtaaaaacagctcttcttcaaatctatacattaagacgactcgaaatccacatatcaaatatccgagtgtagtaaacattccaaaaccgtgatggaatggaacaacacttaaaatcgcagtatccggaatgatttgattgccaaaaataggatctctggcatgcgagaatctgacgcaggcagttctatgcggaagggccacacccttaggtaacccagtagatccagaggagttcattatcagtgcaattgttttgtcacgatcaaaggactctggtacaaaatcgtattcattaaaaccgggaggtagatgagatgtgacgaacgtgtacatcgactgaaatccctggtaatccgttttagaatccatgataataattttctggattattggtaattttttttgcacgttcaaaattttttgcaacccctttttggaaacaaacactacggtaggctgcgaaatgttcatactgttgagcaattcacgttcattataaatgtcgttcgcgggcgcaactgcaactccgataaataacgcgcccaacaccggcataaagaattgaagagagttttcactgcatacgacgattctgtgatttgtattcagcccatatcgtttcatagcttctgccaaccgaacggacatttcgaagtattccgcgtacgtgatgttcacctcgatatgtgcatctgtaaaagcaattgttccaggaaccagggcgtatctcttcatagccttatgcagttgctctccagcggttccatcctctagcggatagaatggcgccgggcctttctttatgtttttggcgtcttccatggtgGCGGCCGCTTTCTTAGCTGCCTATACGGCAGTGAACaagtataagacaaaagtgttgtggaattgctccaggcgatctgacggttcactaaacgagctctgcttttataggcgccCACCGTACACGCCTAAAGCTTATACGTtctctatcactgatagggagtaaactggatatacgttctctatcactgatagggagtaaactgtagatacgttctctatcactgatagggagtaaactggtcatacgttctctatcactgatagggagtaaactccttatacgttctctatcactgatagggagtaaagtctgcatacgttctctatcactgatagggagtaaactcttcatacgttctctatcactgatagggagtaaactcgaatgaagacgaaATGCATGCGTCAATTTTACGCAGACTATCTTTCTAGGGTTAATCTAGCTGCATCAGGATCATATCGTCGGGTCTTTTTTCCGGCTCAGTCATCGCCCAAGCTGGCGCTATCTGGGCATCGGGGAGGAAGAAGCCCGTGCCTTTTCCCGCGAGGTTGAAGCGGCATGGAAAGAGTTTGCCGAGGATGACTGCTGCTGCATTGACGTTGAGCGAAAACGCACGTTTACCATGATGATTCGGGAAGGTGTGGCCATGCACGCCTTTAACGGTGAACTGTTCGTTCAGGCCACCTGGGATACCAGTTCGTCGCGGCTTTTCCGGACACAGTTCCGGATGGTCAGCCCGAAGCGCATCAGCAACCCGAACAATACCGGCGACAGCCGGAACTGCCGTGCCGGTGTGCAGATTAATGACAGCGGTGCGGCGCTGGGATATTACGTCAGCGAGGACGGGTATCCTGGCTGGATGCCGCAGAAATGGACATGGATACCCCGTGAGTTACCCGGCGGGCGCGCTTGGCGTAATCATGGTCATAGCTGTTTCCTGTGTGAAATTGTTATCCGCTCACAATTCCACACAACATACGAGCCGGAAGCATAAAGTGTAAAGCCTGGGGTGCCTAATGAGTGAGCTAACTCACATTAATTGCGTTGCGCTCACTGCCCGCTTTCCAGTCGGGAAACCTGTCGTGCCAGCTGCATTAATGAATCGGCCAACGCGCGGGGAGAGGCGGTTTGCGTATTGGGCGCTCTTCCGCTTCCTCGCTCACTGACTCGCTGCGCTCGGTCGTTCGGCTGCGGCGAGCGGTATCAGCTCACTCAAAGGCGGTAATACGGTTATCCACAGAATCAGGGGATAACGCAGGAAAGAACATGTGAGCAAAAGGCCAGCAAAAGGCCAGGAACCGTAAAAAGGCCGCGTTGCTGGCGTTTTTCCATAGGCTCCGCCCCCCTGACGAGCATCACAAAAATCGACGCTCAAGTCAGAGGTGGCGAAACCCGACAGGACTATAAAGATACCAGGCGTTTCCCCCTGGAAGCTCCCTCGTGCGCTCTCCTGTTCCGACCCTGCCGCTTACCGGATACCTGTCCGCCTTTCTCCCTTCGGGAAGCGTGGCGCTTTCTCATAGCTCACGCTGTAGGTATCTCAGTTCGGTGTAGGTCGTTCGCTCCAAGCTGGGCTGTGTGCACGAACCCCCCGTTCAGCCCGACCGCTGCGCCTTATCCGGTAACTATCGTCTTGAGTCCAACCCGGTAAGACACGACTTATCGCCACTGGCAGCAGCCACTGGTAACAGGATTAGCAGAGCGAGGTATGTAGGCGGTGCTACAGAGTTCTTGAAGTGGTGGCCTAACTACGGCTACACTAGAAGGACAGTATTTGGTATCTGCGCTCTGCTGAAGCCAGTTACCTTCGGAAAAAGAGTTGGTAGCTCTTGATCCGGCAAACAAACCACCGCTGGTAGCGGTGGTTTTTTTGTTTGCAAGCAGCAGATTACGCGCAGAAAAAAAGGATCTCAAGAAGATCCTTTGATCTTTTCTACGGGGTCTGACGCTCAGTGGAACGAAAACTCACGTTAAGGGATTTTGGTCATGAGATTATCAAAAAGGATCTTCACCTAGATCCTTTTAAATTAAAAATGAAGTTTTAAATCAATCTAAAGTATATATGAGTAAACTTGGTCTGACAGTTACCAATGCTTAATCAGTGAGGCACCTATCTCAGCGATCTGTCTATTTCGTTCATCCATAGTTGCCTGACTCCCCGTCGTGTAGATAACTACGATACGGGAGGGCTTACCATCTGGCCCCAGTGCTGCAATGATACCGCGAGACCCACGCTCACCGGCTCCAGATTTATCAGCAATAAACCAGCCAGCCGGAAGGGCCGAGCGCAGAAGTGGTCCTGCAACTTTATCCGCCTCCATCCAGTCTATTAATTGTTGCCGGGAAGCTAGAGTAAGTAGTTCGCCAGTTAATAGTTTGCGCAACGTTGTTGCCATTGCTACAGGCATCGTGGTGTCACGCTCGTCGTTTGGTATGGCTTCATTCAGCTCCGGTTCCCAACGATCAAGGCGAGTTACATGATCCCCCATGTTGTGCAAAAAAGCGGTTAGCTCCTTCGGTCCTCCGATCGTTGTCAGAAGTAAGTTGGCCGCAGTGTTATCACTCATGGTTATGGCAGCACTGCATAATTCTCTTACTGTCATGCCATCCGTAAGATGCTTTTCTGTGACTGGTGAGTACTCAACCAAGTCATTCTGAGAATAGTGTATGCGGCGACCGAGTTGCTCTTGCCCGGCGTCAATACGGGATAATACCGCGCCACATAGCAGAACTTTAAAAGTGCTCATCATTGGAAAACGTTCTTCGGGGCGAAAACTCTCAAGGATCTTACCGCTGTTGAGATCCAGTTCGATGTAACCCACTCGTGCACCCAACTGATCTTCAGCATCTTTTACTTTCACCAGCGTTTCTGGGTGAGCAAAAACAGGAAGGCAAAATGCCGCAAAAAAGGGAATAAGGGCGACACGGAAATGTTGAATACTCATACTCTTCCTTTTTCAATATTATTGAAGCATTTATCAGGGTTATTGTCTCATGAGCGGATACATATTTGAATGTATTTAGAAAAATAAACAAATAGGGGTTCCGCGCACATTTCCCCGAAAAGTGCCAC

plasmid 10

CTAAATTGTAAGCGTTAATATTTTGTTAAAATTCGCGTTAAATTTTTGTTAAATCAGCTCATTTTTTAACCAATAGGCCGAAATCGGCAAAATCCCTTATAAATCAAAAGAATAGACCGAGATAGGGTTGAGTGTTGTTCCAGTTTGGAACAAGAGTCCACTATTAAAGAACGTGGACTCCAACGTCAAAGGGCGAAAAACCGTCTATCAGGGCGATGGCCCACTACGTGAACCATCACCCTAATCAAGTTTTTTGGGGTCGAGGTGCCGTAAAGCACTAAATCGGAACCCTAAAGGGAGCCCCCGATTTAGAGCTTGACGGGGAAAGCCGGCGAACGTGGCGAGAAAGGAAGGGAAGAAAGCGAAAGGAGCGGGCGCTAGGGCGCTGGCAAGTGTAGCGGTCACGCTGCGCGTAACCACCACACCCGCCGCGCTTAATGCGCCGCTACAGGGCGCGTCCCATTCGCCATTCAGGCTGCGCAACTGTTGGGAAGGGCGATCGGTGCGGGCCTCTTCGCTATTACGCCAGCTGGCGAAAGGGGGATGTGCTGCAAGGCGATTAAGTTGGGTAACGCCAGGGTTTTCCCAGTCACGACGTTGTAAAACGACGGCCAGTGAGCGCGCCTCGTTCATTCACGTTTTTGAACCCGTGGAGGACGGGCAGACTCGCGGTGCAAATGTGTTTTACAGCGTGATGGAGCAGATGAAGATGCTCGACACGCTGCAGAACACGCAGCTAGATTAACCCTAGAAAGATAATCATATTGTGACGTACGTTAAAGATAATCATGCGTAAAATTGACGCATGTGTTTTATCGGTCTGTATATCGAGGTTTATTTATTAATTTGAATAGATATTAAGTTTTATTATATTTACACTTACATACTAATAATAAATTCAACAAACAATTTATTTATGTTTATTTATTTATTAAAAAAAAACAAAAACTCAAAATTTCTTCTATAAAGTAACAAAACTTTTATCGAATTttcgtcttcattcgagtttactccctatcagtgatagagaacgtatgaagagtttactccctatcagtgatagagaacgtatgcagactttactccctatcagtgatagagaacgtataaggagtttactccctatcagtgatagagaacgtatgaccagtttactccctatcagtgatagagaacgtatctacagtttactccctatcagtgatagagaacgtatatccagtttactccctatcagtgatagagaACGTATAAGCTTTAGGCGTGTACGGTGggcgcctataaaagcagagctcgtttagtgaaccgtcagatcgcctggagcaattccacaacacttttgtcttatacttAACCAGGGCACGAAACCTCCCTTGGAGAGTCCATAACAAGTAAAGCCTCCTTCTTCGGCGGTAACTTGCAGTGGCCCGTGTCGAATGAAAAGTCTGAAATGCTGCCCCGTACTCTGGGACCGCAGTGTGACAAAAGGCAAGTCAAGGGCGCGGGCCACGGTATCAGGGATTCTTTTTCGGGCCTCTTCCTCGCTAAGATCATGTCGCCGCATGAGTCGCCGTCGCAGCCGTTCTGGATTAGATTTGGCCTGCACTCTAGACACCTGCCGATAAGGGGTTGGGTGGGGAACAACGGCGGGTTCGCCAAACTGCAGGTGATCCCGGAGCCCCTCAAGCCATGGCCTTGCCAGCAAGGCTCTCAGATCGTCGGCAGAAGCGTGGATCCGCAGGCGTTCTCCCAGAATTCAAATGGTGTAATCGCTGTTGTGGGCGGAGCATATTTGTGTATATAAGAGCCCGTGTTAGCTCCTCGATTCAGTCACAAGAGCGCACACACACGCTTATAACTAGCTCTCTCTCTCCACTCAAGGCTAGCGCcaccATGGATCATTATCTGGATATTCGGCTGAGGCCTGATCCAGAGTTCCCACCTGCGCAGCTGATGTCTGTCCTTTTTGGCAAACTTCATCAGGCCCTGGTTGCCCAGGGCGGAGATCGGATAGGGGTAAGCTTTCCAGACCTCGACGAAAGCCGGAGCCGCCTGGGAGAACGCCTGCGGATCCACGCTTCTGCCGACGATCTGAGAGCCTTGCTGGCAAGGCCATGGCTTGAGGGGCTCCGGGATCACCTGCAGTTTGGCGAACCCGCCGTTGTTCCCCACCCAACCCCTTATCGGCAGGTGTCTAGAGTGCAGGCCAAATCTAATCCAGAACGGCTGCGACGGCGACTCATGCGGCGACATGATCTTAGCGAGGAAGAGGCCCGAAAAAGAATCCCTGATACCGTGGCCCGCGCCCTTGACTTGCCTTTTGTCACACTGCGGTCCCAGAGTACGGGGCAGCATTTCAGACTTTTCATTCGACACGGGCCACTGCAAGTTACCGCCGAAGAAGGAGGCTTTACTTGTTATGGACTCTCCAAGGGAGGTTTCGTGCCCTGGTTTtaaGGTACCGGGAGATGGGGGAGGCTAACTGAAACACGGAAGGAGACAATACCGGAAGGAACCCGCGCTATGACGGCAATAAAAAGACAGAATAAAACGCACGGGTGTTGGGTCGTTTGTTCactagtatcaagtgcggaaatttattaaaacacaatttatttcacaatacaattttattaagtaaagttataatgcaagtgtatatagggctacaatatagtatacatggattacaatgtgggtatacaaaagctatagtatggatataaaattattaagcacattggggttacaatataaacataatcaaccaCTTAAGtcacttgtacagctcgtccatgccgccggtggagtggcggccctcggcgcgttcgtactgttccacgatggtgtagtcctcgttgtgggaggtgatgtccaacttgatgttgacgttgtaggcgccgggcagctgcacgggcttcttggccttgtaggtggtcttgacctcagcgtcgtagtggccgccgtccttcagcttcagcctctgcttgatctcgcccttcagggcgccgtcctcggggtacatccgctcggaggaggcctcccagcccatggtTttcttctgcattacggggccgtcggaggggaagttggtgccgcgcagcttcaccttgtagatgaactcgccgtcctgcagggaggagtcctgggtcacggtcaccacgccgccgtcctcgaagttcatcacgcgctcccacttgaagccctcggggaaggacagcttcaagtagtcggggatgtcggcggggtgcttcacgtaggccttggagccgtacatgaactgaggggacaggatgtcccaggcgaagggcagggggccacccttggtcaccttcagcttggcggtctgggtgccctcgtaggggcggccctcgccctcgccctcgatctcgaactcgtggccgttcacggagccctccatgtgcaccttgaagcgcatgaactccttgatgatggccatgttatcctcctcgcccttgctcaccatggtggcaccggtcttgagtggagagagagagctagttataagcgtgtgtgtgcgctcttgtgactgaatcgaggagctaacacgggctcttatatacacaaatatgctccgcccacaacagcgattacaccatttctaggaataaatgagtaacaaggGGATCCcatgatgataaacaatgtatggtgctaatgttgcttcaacaacaattctgttgaactgtgttttcatgtttgccaacaagcacctttatactcggtggcctccccaccaccaacttttttgcactgcaaaaaaacacgcttttgcacgcgggcccatacatagtacaaactctacgtttcgtagactattttacataaatagtctacaccgttgtatacgctccaaatacactaccacacattgaacctttttgcagtgcaaaaaagtacgtgtcggcagtcacgtaggccggccttatcgggtcgcgtcctgtcacgtacgaatcacattatcggaccggacgagtgttgtcttatcgtgacaggacgccagcttcctgtgttgctaaccgcagccggacgcaactccttatcggaacaggacgcgcctccatatcagccgcgcgttatctcatgcgcgtgaccggacacgaggcgcccgtcccgcttatcgcgcctataaatacagcccgcaacgatctggtaaacacagttgaacagcatctgttacagcgacacaacGGTACCgccACCatgtctagactggacaagagcaaagtcataaactctgctctggaattactcaatggagtcggtatcgaaggcctgacgacaaggaaactcgctcaaaagctgggagttgagcagcctaccctgtactggcacgtgaagaacaagcgggccctgctcgatgccctgccaatcgagatgctggacaggcatcatacccactcctgccccctggaaggcgagtcatggcaagactttctgcggaacaacgccaagtcataccgctgtgctctcctctcacatcgcgacggggctaaagtgcatctcggcacccgcccaacagagaaacagtacgaaaccctggaaaatcagctcgcgttcctgtgtcagcaaggcttctccctggagaacgcactgtacgctctgtccgccgtgggccactttacactgggctgcgtattggaggaacaggagcatcaagtagcaaaagaggaaagagagacacctaccaccgattctatgcccccacttctgaaacaagcaattgagctgttcgaccggcagggagccgaacctgccttccttttcggcctggaactaatcatatgtggcctggagaaacagctaaagtgcgaaagcggcgggccgaccgacgcccttgacgattttgacttagacatgctcccagccgatgcccttgacgactttgaccttgatatgctgcctgctgacgctcttgacgattttgaccttgacatgctccccgggtaaGGCGCGCCtttgtattgtcatgttttaatacaatatgttatgtttaaatatgtttttaataaattttataaaataatttcaacttttattgtaacaacattgtccatttacacactcctttcaagcgcgtgcgcacccgaaaagcagggtcgccgctgacgcactgctaaaaatagcacgcgcctttcaaaagcgttcacacccgaaaCTCGAGGTAAGATACATTGATGAGTTTGGACAAACCACAACTAGAATGCAGTGAAAAAAATGCTTTATTTGTGAAATTTGTGATGCTATTGCTTTATTTGTAACCATTATAAGCTGCAATAAACAAGTTAACAACAACAATTGCATTCATTTTATGTTTCAGGTTCAGGGGGAGGTGTGGGAGGTTTTTTAAAGCAAGTAAAACCTCTACAAATGTGGTATGGCTGATTATGATCGTCGACttacaatttggactttccgcccttcttggcctttatgaggatctctctgatttttcttgcgtcgagttttccggtaagacctttcggtacttcgtccacaaacacaactcctccgcgcaactttttggcggttgttacttgactggcgacgtaatccacgatctctttttccgtcatcgtctttccgtgctccaaaacaacaacggcggcgggaagttcaccggcgtcatcgtcgggaagacctgccacgcccgcgtcgaagatgttggggtgttgtaacaatatcgactccaattcagcgggggccacctggtatcctttgtatttaattaaagacttcaagcggtcaactatgaagaagtgttcgtcttcgtcccagtaagctatgtctccagaatgtagccatccatccttgtcaatcaaggcgttggtcgcttccggattgtttacataaccggacataatcataggtcctctgacacataattcgcctctctgattaacgcccagcgttttcccggtatccagatccacaaccttcgcttcaaaaaatggaacaactttaccgaccgcgcccggtttatcatccccctcgggtgtaatcagaatagctgatgtagtctcagtgagcccatatccttgtcgtatccctggaagatggaagcgttttgcaaccgcttccccgacttctttcgaaagaggtgcgcccccagaagcaatttcgtgtaaattagataaatcgtatttgtcaatcagagtgcttttggcgaagaatgaaaatagggttggtactagcaacgcactttgaattttgtaatcctgaagggatcgtaaaaacagctcttcttcaaatctatacattaagacgactcgaaatccacatatcaaatatccgagtgtagtaaacattccaaaaccgtgatggaatggaacaacacttaaaatcgcagtatccggaatgatttgattgccaaaaataggatctctggcatgcgagaatctgacgcaggcagttctatgcggaagggccacacccttaggtaacccagtagatccagaggagttcattatcagtgcaattgttttgtcacgatcaaaggactctggtacaaaatcgtattcattaaaaccgggaggtagatgagatgtgacgaacgtgtacatcgactgaaatccctggtaatccgttttagaatccatgataataattttctggattattggtaattttttttgcacgttcaaaattttttgcaacccctttttggaaacaaacactacggtaggctgcgaaatgttcatactgttgagcaattcacgttcattataaatgtcgttcgcgggcgcaactgcaactccgataaataacgcgcccaacaccggcataaagaattgaagagagttttcactgcatacgacgattctgtgatttgtattcagcccatatcgtttcatagcttctgccaaccgaacggacatttcgaagtattccgcgtacgtgatgttcacctcgatatgtgcatctgtaaaagcaattgttccaggaaccagggcgtatctcttcatagccttatgcagttgctctccagcggttccatcctctagcggatagaatggcgccgggcctttctttatgtttttggcgtcttccatggtgGCGGCCGCTTTCTTAGCTGCCTATACGGCAGTGAACaagtataagacaaaagtgttgtggaattgctccaggcgatctgacggttcactaaacgagctctgcttttataggcgccCACCGTACACGCCTAAAGCTTATACGTtctctatcactgatagggagtaaactggatatacgttctctatcactgatagggagtaaactgtagatacgttctctatcactgatagggagtaaactggtcatacgttctctatcactgatagggagtaaactccttatacgttctctatcactgatagggagtaaagtctgcatacgttctctatcactgatagggagtaaactcttcatacgttctctatcactgatagggagtaaactcgaatgaagacgaaATGCATGCGTCAATTTTACGCAGACTATCTTTCTAGGGTTAATCTAGCTGCATCAGGATCATATCGTCGGGTCTTTTTTCCGGCTCAGTCATCGCCCAAGCTGGCGCTATCTGGGCATCGGGGAGGAAGAAGCCCGTGCCTTTTCCCGCGAGGTTGAAGCGGCATGGAAAGAGTTTGCCGAGGATGACTGCTGCTGCATTGACGTTGAGCGAAAACGCACGTTTACCATGATGATTCGGGAAGGTGTGGCCATGCACGCCTTTAACGGTGAACTGTTCGTTCAGGCCACCTGGGATACCAGTTCGTCGCGGCTTTTCCGGACACAGTTCCGGATGGTCAGCCCGAAGCGCATCAGCAACCCGAACAATACCGGCGACAGCCGGAACTGCCGTGCCGGTGTGCAGATTAATGACAGCGGTGCGGCGCTGGGATATTACGTCAGCGAGGACGGGTATCCTGGCTGGATGCCGCAGAAATGGACATGGATACCCCGTGAGTTACCCGGCGGGCGCGCTTGGCGTAATCATGGTCATAGCTGTTTCCTGTGTGAAATTGTTATCCGCTCACAATTCCACACAACATACGAGCCGGAAGCATAAAGTGTAAAGCCTGGGGTGCCTAATGAGTGAGCTAACTCACATTAATTGCGTTGCGCTCACTGCCCGCTTTCCAGTCGGGAAACCTGTCGTGCCAGCTGCATTAATGAATCGGCCAACGCGCGGGGAGAGGCGGTTTGCGTATTGGGCGCTCTTCCGCTTCCTCGCTCACTGACTCGCTGCGCTCGGTCGTTCGGCTGCGGCGAGCGGTATCAGCTCACTCAAAGGCGGTAATACGGTTATCCACAGAATCAGGGGATAACGCAGGAAAGAACATGTGAGCAAAAGGCCAGCAAAAGGCCAGGAACCGTAAAAAGGCCGCGTTGCTGGCGTTTTTCCATAGGCTCCGCCCCCCTGACGAGCATCACAAAAATCGACGCTCAAGTCAGAGGTGGCGAAACCCGACAGGACTATAAAGATACCAGGCGTTTCCCCCTGGAAGCTCCCTCGTGCGCTCTCCTGTTCCGACCCTGCCGCTTACCGGATACCTGTCCGCCTTTCTCCCTTCGGGAAGCGTGGCGCTTTCTCATAGCTCACGCTGTAGGTATCTCAGTTCGGTGTAGGTCGTTCGCTCCAAGCTGGGCTGTGTGCACGAACCCCCCGTTCAGCCCGACCGCTGCGCCTTATCCGGTAACTATCGTCTTGAGTCCAACCCGGTAAGACACGACTTATCGCCACTGGCAGCAGCCACTGGTAACAGGATTAGCAGAGCGAGGTATGTAGGCGGTGCTACAGAGTTCTTGAAGTGGTGGCCTAACTACGGCTACACTAGAAGGACAGTATTTGGTATCTGCGCTCTGCTGAAGCCAGTTACCTTCGGAAAAAGAGTTGGTAGCTCTTGATCCGGCAAACAAACCACCGCTGGTAGCGGTGGTTTTTTTGTTTGCAAGCAGCAGATTACGCGCAGAAAAAAAGGATCTCAAGAAGATCCTTTGATCTTTTCTACGGGGTCTGACGCTCAGTGGAACGAAAACTCACGTTAAGGGATTTTGGTCATGAGATTATCAAAAAGGATCTTCACCTAGATCCTTTTAAATTAAAAATGAAGTTTTAAATCAATCTAAAGTATATATGAGTAAACTTGGTCTGACAGTTACCAATGCTTAATCAGTGAGGCACCTATCTCAGCGATCTGTCTATTTCGTTCATCCATAGTTGCCTGACTCCCCGTCGTGTAGATAACTACGATACGGGAGGGCTTACCATCTGGCCCCAGTGCTGCAATGATACCGCGAGACCCACGCTCACCGGCTCCAGATTTATCAGCAATAAACCAGCCAGCCGGAAGGGCCGAGCGCAGAAGTGGTCCTGCAACTTTATCCGCCTCCATCCAGTCTATTAATTGTTGCCGGGAAGCTAGAGTAAGTAGTTCGCCAGTTAATAGTTTGCGCAACGTTGTTGCCATTGCTACAGGCATCGTGGTGTCACGCTCGTCGTTTGGTATGGCTTCATTCAGCTCCGGTTCCCAACGATCAAGGCGAGTTACATGATCCCCCATGTTGTGCAAAAAAGCGGTTAGCTCCTTCGGTCCTCCGATCGTTGTCAGAAGTAAGTTGGCCGCAGTGTTATCACTCATGGTTATGGCAGCACTGCATAATTCTCTTACTGTCATGCCATCCGTAAGATGCTTTTCTGTGACTGGTGAGTACTCAACCAAGTCATTCTGAGAATAGTGTATGCGGCGACCGAGTTGCTCTTGCCCGGCGTCAATACGGGATAATACCGCGCCACATAGCAGAACTTTAAAAGTGCTCATCATTGGAAAACGTTCTTCGGGGCGAAAACTCTCAAGGATCTTACCGCTGTTGAGATCCAGTTCGATGTAACCCACTCGTGCACCCAACTGATCTTCAGCATCTTTTACTTTCACCAGCGTTTCTGGGTGAGCAAAAACAGGAAGGCAAAATGCCGCAAAAAAGGGAATAAGGGCGACACGGAAATGTTGAATACTCATACTCTTCCTTTTTCAATATTATTGAAGCATTTATCAGGGTTATTGTCTCATGAGCGGATACATATTTGAATGTATTTAGAAAAATAAACAAATAGGGGTTCCGCGCACATTTCCCCGAAAAGTGCCAC

plasmid 11

CTAAATTGTAAGCGTTAATATTTTGTTAAAATTCGCGTTAAATTTTTGTTAAATCAGCTCATTTTTTAACCAATAGGCCGAAATCGGCAAAATCCCTTATAAATCAAAAGAATAGACCGAGATAGGGTTGAGTGTTGTTCCAGTTTGGAACAAGAGTCCACTATTAAAGAACGTGGACTCCAACGTCAAAGGGCGAAAAACCGTCTATCAGGGCGATGGCCCACTACGTGAACCATCACCCTAATCAAGTTTTTTGGGGTCGAGGTGCCGTAAAGCACTAAATCGGAACCCTAAAGGGAGCCCCCGATTTAGAGCTTGACGGGGAAAGCCGGCGAACGTGGCGAGAAAGGAAGGGAAGAAAGCGAAAGGAGCGGGCGCTAGGGCGCTGGCAAGTGTAGCGGTCACGCTGCGCGTAACCACCACACCCGCCGCGCTTAATGCGCCGCTACAGGGCGCGTCCCATTCGCCATTCAGGCTGCGCAACTGTTGGGAAGGGCGATCGGTGCGGGCCTCTTCGCTATTACGCCAGCTGGCGAAAGGGGGATGTGCTGCAAGGCGATTAAGTTGGGTAACGCCAGGGTTTTCCCAGTCACGACGTTGTAAAACGACGGCCAGTGAGCGCGCCTCGTTCATTCACGTTTTTGAACCCGTGGAGGACGGGCAGACTCGCGGTGCAAATGTGTTTTACAGCGTGATGGAGCAGATGAAGATGCTCGACACGCTGCAGAACACGCAGCTAGATTAACCCTAGAAAGATAATCATATTGTGACGTACGTTAAAGATAATCATGCGTAAAATTGACGCATGTGTTTTATCGGTCTGTATATCGAGGTTTATTTATTAATTTGAATAGATATTAAGTTTTATTATATTTACACTTACATACTAATAATAAATTCAACAAACAATTTATTTATGTTTATTTATTTATTAAAAAAAAACAAAAACTCAAAATTTCTTCTATAAAGTAACAAAACTTTTATCGAATTttcgtcttcattcgagtttactccctatcagtgatagagaacgtatgaagagtttactccctatcagtgatagagaacgtatgcagactttactccctatcagtgatagagaacgtataaggagtttactccctatcagtgatagagaacgtatgaccagtttactccctatcagtgatagagaacgtatctacagtttactccctatcagtgatagagaacgtatatccagtttactccctatcagtgatagagaACGTATAAGCTTTAGGCGTGTACGGTGggcgcctataaaagcagagctcgtttagtgaaccgtcagatcgcctggagcaattccacaacacttttgtcttatacttAACCAGGGCACGAAACCTCCCTTGGAGAGTCCATAACAAGTAAAGCCTCCTTCTTCGGCGGTAACTTGCAGTGGCCCGTGTCGAATGAAAAGTCTGAAATGCTGCCCCGTACTCTGGGACCGCAGTGTGACAAAAGGCAAGTCAAGGGCGCGGGCCACGGTATCAGGGATTCTTTTTCGGGCCTCTTCCTCGCTAAGATCATGTCGCCGCATGAGTCGCCGTCGCAGCCGTTCTGGATTAGATTTGGCCTGCACTCTAGACACCTGCCGATAAGGGGTTGGGTGGGGAACAACGGCGGGTTCGCCAAACTGCAGGTGATCCCGGAGCCCCTCAAGCCATGGCCTTGCCAGCAAGGCTCTCAGATCGTCGGCAGAAGCGTGGATCCGCAGGCGTTCTCCCAGAATTCAAATGGTGTAATCGCTGTTGTGGGCGGAGCATATTTGTGTATATAAGAGCCCGTGTTAGCTCCTCGATTCAGTCACAAGAGCGCACACACACGCTTATAACTAGCTCTCTCTCTCCACTCAAGGCTAGCGCcaccATGGATCATTATCTGGATATTCGGCTGAGGCCTGATCCAGAGTTCCCACCTGCGCAGCTGATGTCTGTCCTTTTTGGCAAACTTCATCAGGCCCTGGTTGCCCAGGGCGGAGATCGGATAGGGGTAAGCTTTCCAGACCTCGACGAAAGCCGGAGCCGCCTGGGAGAACGCCTGCGGATCCACGCTTCTGCCGACGATCTGAGAGCCTTGCTGGCAAGGCCATGGCTTGAGGGGCTCCGGGATCACCTGCAGTTTGGCGAACCCGCCGTTGTTCCCCACCCAACCCCTTATCGGCAGGTGTCTAGAGTGCAGGCCAAATCTAATCCAGAACGGCTGCGACGGCGACTCATGCGGCGACATGATCTTAGCGAGGAAGAGGCCCGAAAAAGAATCCCTGATACCGTGGCCCGCGCCCTTGACTTGCCTTTTGTCACACTGCGGTCCCAGAGTACGGGGCAGCATTTCAGACTTTTCATTCGACACGGGCCACTGCAAGTTACCGCCGAAGAAGGAGGCTTTACTTGTTATGGACTCTCCAAGGGAGGTTTCGTGCCCTGGTTTtaaGGTACCGGGAGATGGGGGAGGCTAACTGAAACACGGAAGGAGACAATACCGGAAGGAACCCGCGCTATGACGGCAATAAAAAGACAGAATAAAACGCACGGGTGTTGGGTCGTTTGTTCactagtatcaagtgcggaaatttattaaaacacaatttatttcacaatacaattttattaagtaaagttataatgcaagtgtatatagggctacaatatagtatacatggattacaatgtgggtatacaaaagctatagtatggatataaaattattaagcacattggggttacaatataaacataatcaaccaCTTAAGtcacttgtacagctcgtccatgccgccggtggagtggcggccctcggcgcgttcgtactgttccacgatggtgtagtcctcgttgtgggaggtgatgtccaacttgatgttgacgttgtaggcgccgggcagctgcacgggcttcttggccttgtaggtggtcttgacctcagcgtcgtagtggccgccgtccttcagcttcagcctctgcttgatctcgcccttcagggcgccgtcctcggggtacatccgctcggaggaggcctcccagcccatggtTttcttctgcattacggggccgtcggaggggaagttggtgccgcgcagcttcaccttgtagatgaactcgccgtcctgcagggaggagtcctgggtcacggtcaccacgccgccgtcctcgaagttcatcacgcgctcccacttgaagccctcggggaaggacagcttcaagtagtcggggatgtcggcggggtgcttcacgtaggccttggagccgtacatgaactgaggggacaggatgtcccaggcgaagggcagggggccacccttggtcaccttcagcttggcggtctgggtgccctcgtaggggcggccctcgccctcgccctcgatctcgaactcgtggccgttcacggagccctccatgtgcaccttgaagcgcatgaactccttgatgatggccatgttatcctcctcgcccttgctcaccatggtggcaccggtcttgagtggagagagagagctagttataagcgtgtgtgtgcgctcttgtgactgaatcgaggagctaacacgggctcttatatacacaaatatgctccgcccacaacagcgattacaccatttctaggaataaatgagtaacaaggGGATCCcatgatgataaacaatgtatggtgctaatgttgcttcaacaacaattctgttgaactgtgttttcatgtttgccaacaagcacctttatactcggtggcctccccaccaccaacttttttgcactgcaaaaaaacacgcttttgcacgcgggcccatacatagtacaaactctacgtttcgtagactattttacataaatagtctacaccgttgtatacgctccaaatacactaccacacattgaacctttttgcagtgcaaaaaagtacgtgtcggcagtcacgtaggccggccttatcgggtcgcgtcctgtcacgtacgaatcacattatcggaccggacgagtgttgtcttatcgtgacaggacgccagcttcctgtgttgctaaccgcagccggacgcaactccttatcggaacaggacgcgcctccatatcagccgcgcgttatctcatgcgcgtgaccggacacgaggcgcccgtcccgcttatcgcgcctataaatacagcccgcaacgatctggtaaacacagttgaacagcatctgttacagcgacacaacGGTACCgccACCatgtctagactggacaagagcaaagtcataaactctgctctggaattactcaatggagtcggtatcgaaggcctgacgacaaggaaactcgctcaaaagctgggagttgagcagcctaccctgtactggcacgtgaagaacaagcgggccctgctcgatgccctgccaatcgagatgctggacaggcatcatacccactcctgccccctggaaggcgagtcatggcaagactttctgcggaacaacgccaagtcataccgctgtgctctcctctcacatcgcgacggggctaaagtgcatctcggcacccgcccaacagagaaacagtacgaaaccctggaaaatcagctcgcgttcctgtgtcagcaaggcttctccctggagaacgcactgtacgctctgtccgccgtgggccactttacactgggctgcgtattggaggaacaggagcatcaagtagcaaaagaggaaagagagacacctaccaccgattctatgcccccacttctgaaacaagcaattgagctgttcgaccggcagggagccgaacctgccttccttttcggcctggaactaatcatatgtggcctggagaaacagctaaagtgcgaaagcggcgggccgaccgacgcccttgacgattttgacttagacatgctcccagccgatgcccttgacgactttgaccttgatatgctgcctgctgacgctcttgacgattttgaccttgacatgctccccgggtaaGGCGCGCCtttgtattgtcatgttttaatacaatatgttatgtttaaatatgtttttaataaattttataaaataatttcaacttttattgtaacaacattgtccatttacacactcctttcaagcgcgtgcgcacccgaaaagcagggtcgccgctgacgcactgctaaaaatagcacgcgcctttcaaaagcgttcacacccgaaaCTCGAGGTAAGATACATTGATGAGTTTGGACAAACCACAACTAGAATGCAGTGAAAAAAATGCTTTATTTGTGAAATTTGTGATGCTATTGCTTTATTTGTAACCATTATAAGCTGCAATAAACAAGTTAACAACAACAATTGCATTCATTTTATGTTTCAGGTTCAGGGGGAGGTGTGGGAGGTTTTTTAAAGCAAGTAAAACCTCTACAAATGTGGTATGGCTGATTATGATCGTCGACtcaagtcagaatagtggacaggcggccaagaacttcgttcatgatagtctccggaacccgttcgagtcgttttccgccccgtgctttcatatcaattgtccggggttgatcgcaacgtacaacacctgtggtacgtatgccaacaccatccaacgacaccgcaaagccggcagtgcgggcaaaattgcctccgctggttacgggcacaacaacaggcaggcgggtcacgcgattaaaggccgccggtgtgacaatcagcaccggccgcgttccctgctgctcatgacctgcggtaggatcaagcgagacaagccagatttcccctctttccatggtgGCGGCCGCTTTCTTAGCTGCCTATACGGCAGTGAACaagtataagacaaaagtgttgtggaattgctccaggcgatctgacggttcactaaacgagctctgcttttataggcgccCACCGTACACGCCTAAAGCTTATACGTtctctatcactgatagggagtaaactggatatacgttctctatcactgatagggagtaaactgtagatacgttctctatcactgatagggagtaaactggtcatacgttctctatcactgatagggagtaaactccttatacgttctctatcactgatagggagtaaagtctgcatacgttctctatcactgatagggagtaaactcttcatacgttctctatcactgatagggagtaaactcgaatgaagacgaaATGCATGCGTCAATTTTACGCAGACTATCTTTCTAGGGTTAATCTAGCTGCATCAGGATCATATCGTCGGGTCTTTTTTCCGGCTCAGTCATCGCCCAAGCTGGCGCTATCTGGGCATCGGGGAGGAAGAAGCCCGTGCCTTTTCCCGCGAGGTTGAAGCGGCATGGAAAGAGTTTGCCGAGGATGACTGCTGCTGCATTGACGTTGAGCGAAAACGCACGTTTACCATGATGATTCGGGAAGGTGTGGCCATGCACGCCTTTAACGGTGAACTGTTCGTTCAGGCCACCTGGGATACCAGTTCGTCGCGGCTTTTCCGGACACAGTTCCGGATGGTCAGCCCGAAGCGCATCAGCAACCCGAACAATACCGGCGACAGCCGGAACTGCCGTGCCGGTGTGCAGATTAATGACAGCGGTGCGGCGCTGGGATATTACGTCAGCGAGGACGGGTATCCTGGCTGGATGCCGCAGAAATGGACATGGATACCCCGTGAGTTACCCGGCGGGCGCGCTTGGCGTAATCATGGTCATAGCTGTTTCCTGTGTGAAATTGTTATCCGCTCACAATTCCACACAACATACGAGCCGGAAGCATAAAGTGTAAAGCCTGGGGTGCCTAATGAGTGAGCTAACTCACATTAATTGCGTTGCGCTCACTGCCCGCTTTCCAGTCGGGAAACCTGTCGTGCCAGCTGCATTAATGAATCGGCCAACGCGCGGGGAGAGGCGGTTTGCGTATTGGGCGCTCTTCCGCTTCCTCGCTCACTGACTCGCTGCGCTCGGTCGTTCGGCTGCGGCGAGCGGTATCAGCTCACTCAAAGGCGGTAATACGGTTATCCACAGAATCAGGGGATAACGCAGGAAAGAACATGTGAGCAAAAGGCCAGCAAAAGGCCAGGAACCGTAAAAAGGCCGCGTTGCTGGCGTTTTTCCATAGGCTCCGCCCCCCTGACGAGCATCACAAAAATCGACGCTCAAGTCAGAGGTGGCGAAACCCGACAGGACTATAAAGATACCAGGCGTTTCCCCCTGGAAGCTCCCTCGTGCGCTCTCCTGTTCCGACCCTGCCGCTTACCGGATACCTGTCCGCCTTTCTCCCTTCGGGAAGCGTGGCGCTTTCTCATAGCTCACGCTGTAGGTATCTCAGTTCGGTGTAGGTCGTTCGCTCCAAGCTGGGCTGTGTGCACGAACCCCCCGTTCAGCCCGACCGCTGCGCCTTATCCGGTAACTATCGTCTTGAGTCCAACCCGGTAAGACACGACTTATCGCCACTGGCAGCAGCCACTGGTAACAGGATTAGCAGAGCGAGGTATGTAGGCGGTGCTACAGAGTTCTTGAAGTGGTGGCCTAACTACGGCTACACTAGAAGGACAGTATTTGGTATCTGCGCTCTGCTGAAGCCAGTTACCTTCGGAAAAAGAGTTGGTAGCTCTTGATCCGGCAAACAAACCACCGCTGGTAGCGGTGGTTTTTTTGTTTGCAAGCAGCAGATTACGCGCAGAAAAAAAGGATCTCAAGAAGATCCTTTGATCTTTTCTACGGGGTCTGACGCTCAGTGGAACGAAAACTCACGTTAAGGGATTTTGGTCATGAGATTATCAAAAAGGATCTTCACCTAGATCCTTTTAAATTAAAAATGAAGTTTTAAATCAATCTAAAGTATATATGAGTAAACTTGGTCTGACAGTTACCAATGCTTAATCAGTGAGGCACCTATCTCAGCGATCTGTCTATTTCGTTCATCCATAGTTGCCTGACTCCCCGTCGTGTAGATAACTACGATACGGGAGGGCTTACCATCTGGCCCCAGTGCTGCAATGATACCGCGAGACCCACGCTCACCGGCTCCAGATTTATCAGCAATAAACCAGCCAGCCGGAAGGGCCGAGCGCAGAAGTGGTCCTGCAACTTTATCCGCCTCCATCCAGTCTATTAATTGTTGCCGGGAAGCTAGAGTAAGTAGTTCGCCAGTTAATAGTTTGCGCAACGTTGTTGCCATTGCTACAGGCATCGTGGTGTCACGCTCGTCGTTTGGTATGGCTTCATTCAGCTCCGGTTCCCAACGATCAAGGCGAGTTACATGATCCCCCATGTTGTGCAAAAAAGCGGTTAGCTCCTTCGGTCCTCCGATCGTTGTCAGAAGTAAGTTGGCCGCAGTGTTATCACTCATGGTTATGGCAGCACTGCATAATTCTCTTACTGTCATGCCATCCGTAAGATGCTTTTCTGTGACTGGTGAGTACTCAACCAAGTCATTCTGAGAATAGTGTATGCGGCGACCGAGTTGCTCTTGCCCGGCGTCAATACGGGATAATACCGCGCCACATAGCAGAACTTTAAAAGTGCTCATCATTGGAAAACGTTCTTCGGGGCGAAAACTCTCAAGGATCTTACCGCTGTTGAGATCCAGTTCGATGTAACCCACTCGTGCACCCAACTGATCTTCAGCATCTTTTACTTTCACCAGCGTTTCTGGGTGAGCAAAAACAGGAAGGCAAAATGCCGCAAAAAAGGGAATAAGGGCGACACGGAAATGTTGAATACTCATACTCTTCCTTTTTCAATATTATTGAAGCATTTATCAGGGTTATTGTCTCATGAGCGGATACATATTTGAATGTATTTAGAAAAATAAACAAATAGGGGTTCCGCGCACATTTCCCCGAAAAGTGCCAC

pxl-BacII-Rluc

CTAAATTGTAAGCGTTAATATTTTGTTAAAATTCGCGTTAAATTTTTGTTAAATCAGCTCATTTTTTAACCAATAGGCCGAAATCGGCAAAATCCCTTATAAATCAAAAGAATAGACCGAGATAGGGTTGAGTGTTGTTCCAGTTTGGAACAAGAGTCCACTATTAAAGAACGTGGACTCCAACGTCAAAGGGCGAAAAACCGTCTATCAGGGCGATGGCCCACTACGTGAACCATCACCCTAATCAAGTTTTTTGGGGTCGAGGTGCCGTAAAGCACTAAATCGGAACCCTAAAGGGAGCCCCCGATTTAGAGCTTGACGGGGAAAGCCGGCGAACGTGGCGAGAAAGGAAGGGAAGAAAGCGAAAGGAGCGGGCGCTAGGGCGCTGGCAAGTGTAGCGGTCACGCTGCGCGTAACCACCACACCCGCCGCGCTTAATGCGCCGCTACAGGGCGCGTCCCATTCGCCATTCAGGCTGCGCAACTGTTGGGAAGGGCGATCGGTGCGGGCCTCTTCGCTATTACGCCAGCTGGCGAAAGGGGGATGTGCTGCAAGGCGATTAAGTTGGGTAACGCCAGGGTTTTCCCAGTCACGACGTTGTAAAACGACGGCCAGTGAGCGCGCCTCGTTCATTCACGTTTTTGAACCCGTGGAGGACGGGCAGACTCGCGGTGCAAATGTGTTTTACAGCGTGATGGAGCAGATGAAGATGCTCGACACGCTGCAGAACACGCAGCTAGATTAACCCTAGAAAGATAATCATATTGTGACGTACGTTAAAGATAATCATGCGTAAAATTGACGCATGTGTTTTATCGGTCTGTATATCGAGGTTTATTTATTAATTTGAATAGATATTAAGTTTTATTATATTTACACTTACATACTAATAATAAATTCAACAAACAATTTATTTATGTTTATTTATTTATTAAAAAAAAACAAAAACTCAAAATTTCTTCTATAAAGTAACAAAACTTTTATCGAATTCCTGCAGCCCGGGGGATCCccttgttactcatttattcctagaaatggtgtaatcgctgttgtgggcggagcatatttgtgtatataagagcccgtgttagctcctcgattcagtcacaagagcgcacacacacgcttataactagctctctctctccactcaagGGTACCACCatggcttccaaggtgtacgaccccgagcaacgcaaacgcatgatcactgggcctcagtggtgggctcgctgcaagcaaatgaacgtgctggactccttcatcaactactatgattccgagaagcacgccgagaacgccgtgatttttctgcatggtaacgctgcctccagctacctgtggaggcacgtcgtgcctcacatcgagcccgtggctagatgcatcatccctgatctgatcggaatgggtaagtccggcaagagcgggaatggctcatatcgcctcctggatcactacaagtacctcaccgcttggttcgagctgctgaaccttccaaagaaaatcatctttgtgggccacgactggggggcttgtctggcctttcactactcctacgagcaccaagacaagatcaaggccatcgtccatgctgagagtgtcgtggacgtgatcgagtcctgggacgagtggcctgacatcgaggaggatatcgccctgatcaagagcgaagagggcgagaaaatggtgcttgagaataacttcttcgtcgagaccatgctcccaagcaagatcatgcggaaactggagcctgaggagttcgctgcctacctggagccattcaaggagaagggcgaggttagacggcctaccctctcctggcctcgcgagatccctctcgttaagggaggcaagcccgacgtcgtccagattgtccgcaactacaacgcctaccttcgggccagcgacgatctgcctaagatgttcatcgagtccgaccctgggttcttttccaacgctattgtcgagggagctaagaagttccctaacaccgagttcgtgaaggtgaagggcctccacttcagccaggaggacgctccagatgaaatgggtaagtacatcaagagcttcgtggagcgcgtgctgaagaacgagcagtaaGGCGCGCCGATCATAATCAGCCATACCACATTTGTAGAGGTTTTACTTGCTTTAAAAAACCTCCCACACCTCCCCCTGAACCTGAAACATAAAATGAATGCAATTGTTGTTGTTAACTTGTTTATTGCAGCTTATAATGGTTACAAATAAAGCAATAGCATCACAAATTTCACAAATAAAGCATTTTTTTCACTGCATTCTAGTTGTGGTTTGTCCAAACTCATCAATGTATCTTATCATGTCTGGATCTCGAGGTCGACGGTATCGATAAGCTTGATATCTATAACAAGAAAATATATATATAATAAGTTATCACGTAAGTAGAACATGAAATAACAATATAATTATCGTATGAGTTAAATCTTAAAAGTCACGTAAAAGATAATCATGCGTCATTTTGACTCACGCGGTCGTTATAGTTCAAAATCAGTGACACTTACCGCATTGACAAGCACGCCTCACGGGAGCTCCAAGCGGCGACTGAGATGTCCTAAATGCACAGCGACGGATTCGCGCTATTTAGAAAGAGAGAGCAATATTTCAAGAATGCATGCGTCAATTTTACGCAGACTATCTTTCTAGGGTTAATCTAGCTGCATCAGGATCATATCGTCGGGTCTTTTTTCCGGCTCAGTCATCGCCCAAGCTGGCGCTATCTGGGCATCGGGGAGGAAGAAGCCCGTGCCTTTTCCCGCGAGGTTGAAGCGGCATGGAAAGAGTTTGCCGAGGATGACTGCTGCTGCATTGACGTTGAGCGAAAACGCACGTTTACCATGATGATTCGGGAAGGTGTGGCCATGCACGCCTTTAACGGTGAACTGTTCGTTCAGGCCACCTGGGATACCAGTTCGTCGCGGCTTTTCCGGACACAGTTCCGGATGGTCAGCCCGAAGCGCATCAGCAACCCGAACAATACCGGCGACAGCCGGAACTGCCGTGCCGGTGTGCAGATTAATGACAGCGGTGCGGCGCTGGGATATTACGTCAGCGAGGACGGGTATCCTGGCTGGATGCCGCAGAAATGGACATGGATACCCCGTGAGTTACCCGGCGGGCGCGCTTGGCGTAATCATGGTCATAGCTGTTTCCTGTGTGAAATTGTTATCCGCTCACAATTCCACACAACATACGAGCCGGAAGCATAAAGTGTAAAGCCTGGGGTGCCTAATGAGTGAGCTAACTCACATTAATTGCGTTGCGCTCACTGCCCGCTTTCCAGTCGGGAAACCTGTCGTGCCAGCTGCATTAATGAATCGGCCAACGCGCGGGGAGAGGCGGTTTGCGTATTGGGCGCTCTTCCGCTTCCTCGCTCACTGACTCGCTGCGCTCGGTCGTTCGGCTGCGGCGAGCGGTATCAGCTCACTCAAAGGCGGTAATACGGTTATCCACAGAATCAGGGGATAACGCAGGAAAGAACATGTGAGCAAAAGGCCAGCAAAAGGCCAGGAACCGTAAAAAGGCCGCGTTGCTGGCGTTTTTCCATAGGCTCCGCCCCCCTGACGAGCATCACAAAAATCGACGCTCAAGTCAGAGGTGGCGAAACCCGACAGGACTATAAAGATACCAGGCGTTTCCCCCTGGAAGCTCCCTCGTGCGCTCTCCTGTTCCGACCCTGCCGCTTACCGGATACCTGTCCGCCTTTCTCCCTTCGGGAAGCGTGGCGCTTTCTCATAGCTCACGCTGTAGGTATCTCAGTTCGGTGTAGGTCGTTCGCTCCAAGCTGGGCTGTGTGCACGAACCCCCCGTTCAGCCCGACCGCTGCGCCTTATCCGGTAACTATCGTCTTGAGTCCAACCCGGTAAGACACGACTTATCGCCACTGGCAGCAGCCACTGGTAACAGGATTAGCAGAGCGAGGTATGTAGGCGGTGCTACAGAGTTCTTGAAGTGGTGGCCTAACTACGGCTACACTAGAAGGACAGTATTTGGTATCTGCGCTCTGCTGAAGCCAGTTACCTTCGGAAAAAGAGTTGGTAGCTCTTGATCCGGCAAACAAACCACCGCTGGTAGCGGTGGTTTTTTTGTTTGCAAGCAGCAGATTACGCGCAGAAAAAAAGGATCTCAAGAAGATCCTTTGATCTTTTCTACGGGGTCTGACGCTCAGTGGAACGAAAACTCACGTTAAGGGATTTTGGTCATGAGATTATCAAAAAGGATCTTCACCTAGATCCTTTTAAATTAAAAATGAAGTTTTAAATCAATCTAAAGTATATATGAGTAAACTTGGTCTGACAGTTACCAATGCTTAATCAGTGAGGCACCTATCTCAGCGATCTGTCTATTTCGTTCATCCATAGTTGCCTGACTCCCCGTCGTGTAGATAACTACGATACGGGAGGGCTTACCATCTGGCCCCAGTGCTGCAATGATACCGCGAGACCCACGCTCACCGGCTCCAGATTTATCAGCAATAAACCAGCCAGCCGGAAGGGCCGAGCGCAGAAGTGGTCCTGCAACTTTATCCGCCTCCATCCAGTCTATTAATTGTTGCCGGGAAGCTAGAGTAAGTAGTTCGCCAGTTAATAGTTTGCGCAACGTTGTTGCCATTGCTACAGGCATCGTGGTGTCACGCTCGTCGTTTGGTATGGCTTCATTCAGCTCCGGTTCCCAACGATCAAGGCGAGTTACATGATCCCCCATGTTGTGCAAAAAAGCGGTTAGCTCCTTCGGTCCTCCGATCGTTGTCAGAAGTAAGTTGGCCGCAGTGTTATCACTCATGGTTATGGCAGCACTGCATAATTCTCTTACTGTCATGCCATCCGTAAGATGCTTTTCTGTGACTGGTGAGTACTCAACCAAGTCATTCTGAGAATAGTGTATGCGGCGACCGAGTTGCTCTTGCCCGGCGTCAATACGGGATAATACCGCGCCACATAGCAGAACTTTAAAAGTGCTCATCATTGGAAAACGTTCTTCGGGGCGAAAACTCTCAAGGATCTTACCGCTGTTGAGATCCAGTTCGATGTAACCCACTCGTGCACCCAACTGATCTTCAGCATCTTTTACTTTCACCAGCGTTTCTGGGTGAGCAAAAACAGGAAGGCAAAATGCCGCAAAAAAGGGAATAAGGGCGACACGGAAATGTTGAATACTCATACTCTTCCTTTTTCAATATTATTGAAGCATTTATCAGGGTTATTGTCTCATGAGCGGATACATATTTGAATGTATTTAGAAAAATAAACAAATAGGGGTTCCGCGCACATTTCCCCGAAAAGTGCCAC
