# Supplementary material for: A low-background Tet-On system based on post-transcriptional regulation using Csy4
Source: PLoS One. 2020 Dec 30;15(12):e0244732. doi: 10.1371/journal.pone.0244732 (PMC7773235; doi:10.1371/journal.pone.0244732)
Supplement: Attachment — Submitted filename: Response to Reviewers.docx [file pone.0244732.s003.docx]

Dear Editors and Reviewers:

Many thanks for the helpful comments on our manuscript. Those comments are all valuable and very helpful for revising and improving our paper We have made revisions based on the comments by the reviewers. Here is our point-by-point response to the reviewers’ comments for the revision.

Kind regards

Corresponding author: Zhihui Zhu

E-mail address: zhihui@mail.hzau.edu.cn

Replies to the reviewers’ comments:

**Reviewer #1:**

This is a potentially interesting approach to reduce the basal level of transgene expression, a frequent drawback to the use of the tetracycline-inducible system.

The authors are using a Crispr/Cas9-analog, csy4, associated with a csy4 target sequence placed between the tetracycline-responsive elements (TRE) to reduce transcription. As expected both basal and induced levels were reduced.

In order to differentially affect the induced and uninduced levels, the authors have an antisense csy4 sequence under the control of a second TRE. Thus, doxycycline treatment presumably resulted in the post-transcriptional blockage of csy4 and increase full expression of the transgene.

However, the study as it is presented falls short in the data justifying the interpretation, lacks important controls and the statistical analysis is not correct.

**Question 1：**

I) No mRNA analysis is provided to support the proposed mechanism.

**Response:** We have added mRNA analysis to support the proposed mechanism (See Fig 3).

**Question 2：**

II) p10 plasmid: no toxicity analysis has been performed. Therefore the hypothesis that kid 1 is the origin of the toxicity observed with p11 in Fig.4 is not proven. The rccsy4 sequence could be toxic as well.

**Response:** Long double-stranded RNA is cytotoxic in mammals, but there is no report that antisense RNA is toxic in insect cells. In insects, long double-stranded RNA is usually used for RNAi. After the p10 plasmid is transfected into sf9 cells, if the antisense csy4 is toxic, the luciferase activity will be affected, and the expression of red fluorescent protein will also be affected. However, no significant changes in red fluorescence after dox-induced expression of rcsy4 are observed (Fig 1). So we think that rcsy4 is not toxic to sf9 cells.

Fig 1: sf9 cells transfected with p1 and p10 plasmids.

p11 plasmid: a demonstration that kid 1 is expressed and its level of dox induction is missing. Again, the only evidence that kid 1 is expressed is indirect and in the absence of an appropriate control, the authors cannot exclude that rccsy4 is toxic.

**Response:** We have tried to use RT-PCR to detect the mRNA expression level of kid gene after induction. Due to the strong toxicity of Kid, the cells that induced the expression of kid died quickly, so it was difficult to detect the expression of kid Gene by RT-PCR. In the last question, we have eliminated the toxicity of rcsy4, so we can indirectly observe the number of red fluorescent cells to determine whether the kid gene is expressed.

**Question 3：**

III) The statistical analysis is not adequate:

a. Fig.2 and Fig.4 a student T test can be performed for 2 groups. For more than 2 groups, a one-way ANOVA analysis should be performed. In Fig.4 a two-way ANOVA analysis could also be performed to take into account the Dox treatment.

**Response:** Thanks for your advice we have performed one-way ANOVA analysis in Fig.2 and Fig.4. After DOX induction, the values changed too much, and two-way ANOVA was not needed. There is no relevant literature reported that DOX induction was considered by two-way ANOVA

b. In Fig.3 there is no statistical analysis.

**Response:** I have checked a lot of related literature on Tet-on system optimization. There is no literature in the related luciferase experiment for statistical analysis of its results. Everyone is concerned about the background reduction level and dox induced changes. (Das, et al. 2016, Forster, et al. 1999, Loew, et al. 2010, Zhou, et al. 2006)

Reference:

1.	Das AT, Tenenbaum L, Berkhout B. Tet-On Systems For Doxycycline-inducible Gene Expression. *Current gene therapy*, 2016, 16(3): 156-167

2.	Forster K, Helbl V, Lederer T, Urlinger S, Wittenburg N, Hillen W. Tetracycline-inducible expression systems with reduced basal activity in mammalian cells. *Nucleic acids research*, 1999, 27(2): 708-710

3.	Loew R, Heinz N, Hampf M, Bujard H, Gossen M. Improved Tet-responsive promoters with minimized background expression. *BMC biotechnology*, 2010, 10: 81

4.	Zhou X, Vink M, Klaver B, Berkhout B, Das AT. Optimization of the Tet-On system for regulated gene expression through viral evolution. *Gene therapy*, 2006, 13(19): 1382-1390

IV) the dox dose is provided only in one case: the luciferase assay. This is a low dose (50ng/ml). Has the same dose been used for all other experiments? A dose range optimization would be interesting.

**Response:** Yes, the same dose has been used in other experiments, and we have added dox dose in other cases, The Tet-on 3G system is an optimized Tet-on system that is highly sensitive to doxycycline. We have tested the induction effect of different concentrations of dox in sf9 cells and found that 50ng/mL has reached the maximum induction effect in sf9 cells. This concentration is consistent with the results reported in previous articles (Loew, et al. 2010). Our main purpose is to study the effect of the csy4 system on the background expression level, so we only selected the maximum induction dose for our experiment.

Reference:

Loew R, Heinz N, Hampf M, Bujard H, Gossen M. Improved Tet-responsive promoters with minimized background expression. *BMC biotechnology*, 2010, 10: 81

**Reviewer #2:**

The manuscript presents data on the development of a method to suppress leaky expression by the Tet-On 3G regulated promoter. The employment of the Tet-On 3G promoter has great utility in providing an accurately regulated expression system for many applications. However, the authors clearly demonstrate that the promoter system does have low level leaky expression that can interfere with the desired activity of the system. The coupling of a highly lethal product, Kid protein, with the promoter results in high levels of background cell death as a marker to measure the amount of low-level background transcription from the Tet-On 3G system. To reduce the background further, the authors employ an additional level of negative regulation on the Tet-On 3G system by utilizing a TRE/ WSSVie1 (Δ23) promotor-driven sense/anti-sense csy4 regulator that reduced the background induced cell death and increasing the induction increase by nearly 400-fold.

**Question 1：**

The authors present their data as luciferase assays and death of fluorescent Sf9 cells. However, the authors do not present any RT-qPCR data on the transcript levels that would support the indirect observations. The RT-qPCR data are critical confirming the transcript reductions reported.

**Response:** Thank you for your advice. We have done RT-qPCR to prove the reduction of transcripts.(See Fig 3)

**Question 2：**

The authors also do not report on the collateral effects on the no-transformed cells – or even the rate of transfection. The authors should have included data on the number of non-transformed cells present use a nuclear stain (DAPI) to assess the impact of collateral effects and toxicities from the Kid protein release from the dying cells.

**Response:** The transfection efficiency of Sf9 cells was about 10%. If we study the toxicity of kid protein to Sf9 cells, it is necessary to study the effect of the kid protein released by death cells on peripheral healthy cells. But our experiment aims to prove that the background expression level of teton3g system is high when teton3g system control the expression of toxic protein. Therefore, the toxic protein released by dead cells affects surrounding healthy cells is beyond the scope of our research.

**Question 3：**

The comments that the small fluorescent bodies in figures 2 and 4 represent cell death are not sufficiently clear since there are other objects that are of the same size and fluorescence that appear to be healthy cells in the field. This has to be clarified either by a live stain or another method to validate the observations.

**Response:** It is a normal phenomenon that there will be a small amount of death in the cultured cells, and the transfection reagent will also be toxic. Therefore, some cell fragments and fluorescents can be observed in healthy cells, but the number is much lower than that of the cells transfected with kid plasmid. We have tried to use live cell staining to distinguish the state of the cells, but the transfection efficiency of insect cell lines is too low. With live cell staining, there is almost no difference between the treatment and the control. This is why we indirectly explain the expression of kid by observing the red fluorescence status of cells.

**Question 4：**

The authors are redundant in the wording of the Results and Discussion. Lines 270 and 348 have “time consuming”, lines 274 and 349 have “unwanted Csy4” showing that the same verbiage has been utilized in both sections.

**Response:** We have changed the same verbiage. (See manuscript lines 285 and 289)

**Question 5：**

Additionally, “harboring” is used in lines 209, 211 and 354 to indicate the presence of a plasmid. “Harboring” means to shelter or hide something which is not the intent of transfecting these plasmids into the Sf9 cells it is to measure activity.

**Response:** Sorry for the errors. We have changed this word. (See lines 226,228 and 369)
