# Supplementary material for: A low-background Tet-On system based on post-transcriptional regulation using Csy4
Source: PLoS One. 2020 Dec 30;15(12):e0244732. doi: 10.1371/journal.pone.0244732 (PMC7773235; doi:10.1371/journal.pone.0244732)
Supplement: Attachment — Submitted filename: Response to Reviewers.docx [file pone.0244732.s004.docx]

Dear Editors and Reviewers:

Many thanks for the helpful comments on our manuscript. Those comments are all valuable and very helpful for revising and improving our paper We hope this version will be acceptable for publication. Here is our point-by-point response to the reviewers’ comments for the revision.

Kind regards

Corresponding author: Zhihui Zhu

E-mail address: [zhihui@mail.hzau.edu.cn](mailto:zhihui@mail.hzau.edu.cn)

Replies to the reviewers’ comments:

**Reviewer #1:**

**Question 1：**

An important concern (question 3b) has not been addressed.

In Fig.3, neither the luciferase mesaures, nor the mRNA quantification have been submitted to a statistical analysis. Regardeless of the type of measure, a relevant statistical analysis is required to validate the conclusion.

**Response:** Thank you for your suggestion. We have made a statistical analysis of the data in Figure 3.

**Question 2：**

In this Figure, data are presented as means +/- standard deviation. It is only mentionned that “Error bars indicate the standard deviation of three replicates.”. What means “three replicates”? Were 3 independent experiments performed or were the measures of on experiment performed in triplicate?

**Response:** We are sorry for this mistake. We mean three independent experiments. We have corrected it in the manuscript. (See line 160)

**Question 3：**

The authors claim that “There is no literature in the related luciferase experiment for statistical analysis of its results”. This is not correct. Among the citations that they provide, Das, et al. 2016 is a review and Loew, et al. 2010 provide a statistical analysis of luciferase measurements. See also Vanrell et al., 2011 {Vanrell, 2011 #592}, and Chtarto et al., 2016 {Chtarto, 2016 #1056} for example.

**Response:** We have checked the literature again, we are sorry for our mistake, we have done statistical analysis. (See Fig. 3)

**Reviewer #2:**

**Question 1:**

The argument that antisense csy4 is not toxic could have been resolved by simply expressing it in tagged cells, however the indirect evidence is acceptable. A qualifying statement about the lack of antisense csy4 toxicity should be included in the results.

**Response:** Thanks for your suggestion. We have added a statement about the toxicity of antisense csy4 to the results. (See lines 295 and 296)

**Question 2:**

The inclusion of the RT-qPCR data in Figure 3 was useful but the statistical analysis of the data was not included. When analyzing the differences, between samples the authors should have applied ANOVA to show that the differences were significant and then complete the analysis using Tuckey’s multiple comparison test to discriminate between the means of selected treatments. Showing only the fold differences between the treatments does not provide assurance of significance.

Similarly, the Tuckey’s MCT should also be used to complete the analysis of the fluorescent cell counts in Figures 2 and 4. The data is such that a statistical analysis is appropriate.

**Response:** Thank you for your comments. We have performed statistical analysis as shown in Figures 2 and 4. (See Figure 3)
